# Fluorinated PAMAM-Arginine Carrier Prodrugs for pH-Sensitive Sustained Ibuprofen Delivery

**DOI:** 10.1007/s11095-024-03747-6

**Published:** 2024-07-24

**Authors:** Carola Romani, Mattia Sponchioni, Alessandro Volonterio

**Affiliations:** 1 Department of Chemistry, Materials and Chemical Engineering “Giulio Natta”, Politecnico Di Milano, Via Mancinelli 7, 20131 Milano, Italy; 2 Consiglio Nazionale delle Ricerche, Istituto di Scienze e Tecnologie Chimiche “Giulio Natta” (SCITEC), Via Mario Bianco 9, 20131 Milan, Italy

**Keywords:** dendrimer, drug delivery system, fluorine, ibuprofen, prodrug

## Abstract

**Objective:**

The development of an efficient, multifunctional drug delivery system overcoming different obstacles generally associated with drug formulations, including the poor accumulation of the active principle in the target site and its sustained release for prolonged time.

**Methods:**

Our study proposes the development of a fluorinated poly(amidoamine) (PAMAM) carrier prodrug combining drug release boosted in alkaline environments with a possible implementation in ^19^F MRI applications. In particular, we functionalized the terminal primary amines of PAMAM G2 and G4 through an ad hoc designed fluorinated ibuprofen-arginine Michael acceptor to obtain multifunctional ibuprofen-PAMAM-Arg conjugates.

**Results:**

These carriers demonstrated pH-dependent and sustained ibuprofen release for more than 5 days. This advantage was observed in both weak alkaline and physiological buffer solutions, allowing to overcome the limits associated to the burst release from similar fluorinated Arg-PAMAM dendrimers with ibuprofen physically encapsulated.

**Conclusion:**

These findings, coupled to the high biocompatibility of the system, suggest a potential synergistic biomedical application of our conjugates, serving as vehicles for drug delivery and as ^19^F magnetic resonance imaging contrast agents.

**Supplementary Information:**

The online version contains supplementary material available at 10.1007/s11095-024-03747-6.

## Introduction

Controlled drug delivery systems are designed to introduce therapeutic substances into the body at appropriate rate, timing, and location. These aims would ultimately overcome the drawbacks associated with the traditional drug formulations, such as the limited blood circulation half-life, reflected in a poor accumulation in the target tissue, a burst drug release, requiring multiple administrations, and cytotoxicity for some of the currently employed excipients [1, 2]. So far, the most investigated drug delivery systems include lipidic or polymeric particles, such as liposomes [3], PEGylated polymeric and/or lipidic vehicles [4, 5], nanoparticles [6–8], and dendrimers [9–11]. In this scenario poly(amidoamine) (PAMAM) dendrimers have shown a great potential as vehicles for pharmacological agents, biomolecules, and peptides and/or as pro-drug systems, thanks to a well-defined branched structure, their terminal functional groups amenable for straightforward functionalization, homogenous charge density distribution, and internal hydrophobic cavity [12–17]. In this direction, the terminal primary amines play a pivotal role in electrostatic interactions and for the potential functionalization of dendritic surfaces. In particular, the functionalization of the outer amines has been often exploited to improve the interactions with the cargo (drugs and/or genes), to decrease cytotoxicity, to promote cellular internalization, and to impart tunable functional properties for different applications, like detectability in magnetic resonance imaging (MRI) [15, 17–20].

In the last decade, different research groups working in the field of cancer therapy and neurodegenerative diseases among others, highlighted the effectiveness and the tunable properties of PAMAM dendrimers in the delivery of hydrophobic and/or anionic drugs, reducing systemic effects and increasing the efficiency at the targeted site before body clearance [9, 10, 20–22]. For this purpose, drug/PAMAM complexes and small drug-dendrimer conjugates are nowadays highly considered and deeply investigated by the scientific community. Drug/dendrimer complexes are formed by non-covalent interactions, such as hydrophobic and electrostatic. This physical drug encapsulation has been widely studied, showing a good capability of PAMAM to encapsulate different types of drugs, from nonsteroidal anti-inflammatory ones such as ibuprofen (IBU) [23] and dexamethasone (DEXA) [24] to anticancer drugs such as doxorubicin (DOX) [25]. In these studies, a strong influence of the dendrimer generation, size, steric hindrance, surface functional groups, and of the degree of PEGylation often exploited to increase the stability of the formulation and to reduce its cytotoxicity was revealed on both drug loading and drug release [10, 26, 27]. However, a critical aspect that is still invariably connected to the use of PAMAM dendrimers as delivery systems is the rapid release of the payload, preventing a sustained drug release for extended periods. This significantly compromises the patient compliance, as multiple administrations of the formulation, typically through parenteral routes, is required [3, 28, 29].

In this scenario, carrier prodrugs, where the drug is covalently tethered to the vehicle, could be an important strategy to alleviate the mentioned drawback of burst release. The prodrug strategy transfers the control on the drug release rate from diffusion phenomena to the kinetic of drug-carrier bond cleavage, which typically occurs with longer characteristic times [25]. This concept can be elevated by considering stimuli-responsive bonds, chemical links between the drug and the carrier that can be cleaved on demand upon application of an external trigger [30, 31].

pH is probably the most investigated stimulus to realize smart prodrugs [1, 32]. The design of pH-responsive vehicles frequently entails linking the drug to a polymeric support using degradable links like ester bonds [33–35], carbamates [36], imine bonds [37], and hydrazones [38, 39]. The careful design of the conjugation strategy is crucial to achieve high drug loading efficiency, enhanced water solubility of hydrophobic drugs, prolonged and controlled release kinetics, and drug release in specific targeted sites, avoiding early release inside the blood stream.

Among several types of polymeric vectors, the use of PAMAM as a multifunctional delivery system has attracted increasing attention. The chemical and structural features of PAMAM dendrimers give them, for example, suitable characteristics to be good candidates for the simultaneous delivery of genes and drugs. Surface grafting with small interfering RNA (siRNA), chemotherapy agents, or anti-inflammatory drugs coupled with drug or gene encapsulation has emerged as a promising strategy in the development of co-delivery systems for treating human diseases [40–43]. This approach, with its synergistic effects, holds potential in fields like cancer therapy and the treatment of conditions such as acute respiratory distress syndrome. Moreover, the integration of innovative fluorinated organic contrast agents appears to play a significant role in breast cancer therapy [20, 41]. In fact, the possibility that fluorine imaging tracers introduce for investigation through ^19^F-MRI or in MR/optical imaging opens up the appealing opportunity to integrate therapy and diagnostic in a single drug delivery carrier.

**Chart 1:**
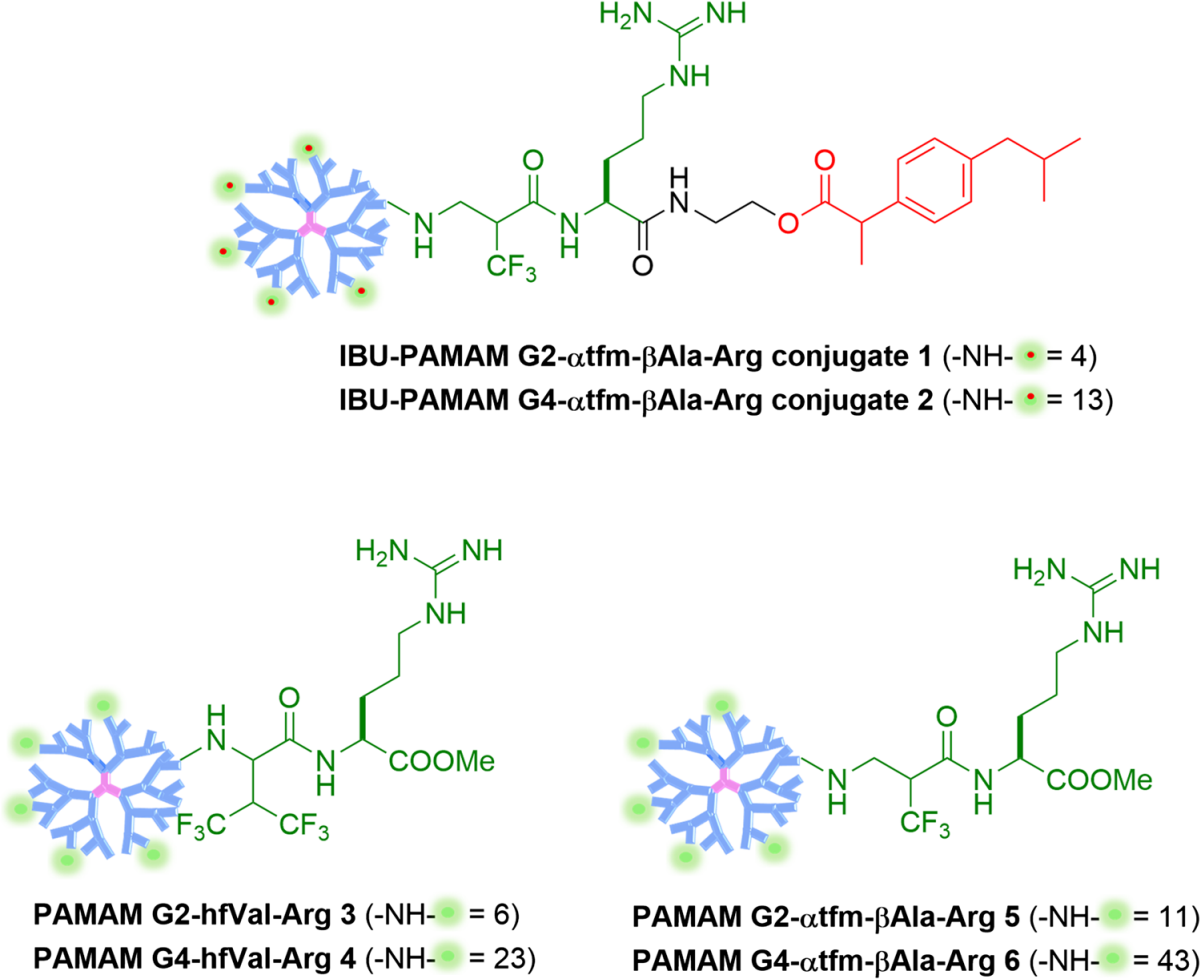
Structures of selectively fluorinated IBU-PAMAM-Arg conjugates **1,2** and PAMAM-Arg conjugates **3–6**^15^.

In this context, we present herein the synthesis and application of multifunctional fluorinated PAMAM-arginine (PAMAM-Arg) conjugates **1–6** for integrated drug delivery and imaging (Chart 1). In particular, we report a drug delivery comparative study, both in terms of grafting/encapsulation of the anti-inflammatory drug IBU and release rate, between novel fluorinated IBU-PAMAM G2- and G4-α-trifluoromethyl-β-alanine-arginine (IBU-PAMAM G2- and G4-α-tfm-β-Ala-Arg) prodrugs **1**–**2**, and fluorinated PAMAM-Arg conjugates, namely PAMAM G2 and PAMAM G4 hfVal-Arg **3–4** and PAMAM G2 and PAMAM G4 α-tfm-β-Ala-Arg **5**–**6** with IBU physically encapsulated. Specifically, in the case of fluorinated PAMAM-Arg conjugates **3–6**, IBU was encapsulated via nanoprecipitation, eliminating the necessity for additional postprocessing steps, as previously documented [44]. On the other side, IBU-PAMAM G2- and G4-α-tfm-β-Ala-Arg **1**–**2** conjugates were synthesized as innovative fluorinated stimuli-responsive prodrug dendrimers with a possible implementation as theragnostic tools, combining drug delivery and detection via fluorine MRI (Chart 1). For this purpose, IBU was conjugated to the vector through an ester bond to obtain a controlled release mediated by its hydrolysis operated by endogenous esterase, mimicked in this work by alkaline pH 8 in drug release experimental trials.

## Materials and Method

### Materials

PAMAM G2 and PAMAM G4 dendrimer 2 (ethylenediamine core, 16 and 64 surface groups respectively, products number: 412406 and 412,449 respectively) and all other organic reactants, solvents and culture reagents were purchased from Sigma-Aldrich (Milan, Italy) if not differently specified and used as received. 2-(Trifluoromethyl)acryloyl chloride **8** was synthesized from 2-(trifluoromethyl)acrylic acid as described in literature [45]. Spectra/Por dialysis bags (MWCO = 1 kDa) were from Spectrum laboratories (Compton, CA, USA). Human Umbilical Vein Endothelial Cells (HUVEC) were purchased from PromoCell (Heidelberg, Germany).

^1^H, ^13^C, and ^19^F NMR spectra were recorded on 400 MHz spectrometers. Chemical shifts are expressed in ppm (δ), using tetramethylsilane (TMS) as internal standard for ^1^H and ^13^C nuclei (δ_H_ and δ_C_ = 0.00) while C_6_F_6_ was used as external standard (δ_F_ − 162.90) for ^19^F. ESI mass spectra were performed by a Bruker Esquire 3000 + instrument equipped with a MS detector composed by a ESI ionization source and a Single Quadrupole mass selective detector. Purifications of the intermediates was performed by Flash Chromatography (FC) with Biotage Isolera One Flash Purification Chromatography ISO-1SV Unit4 Pred Selekt.

### Synthesis of IBU-N^ω^-((2,2,4,6,7-pentamethyl-2,3-dihydrobenzofuran-5-yl)sulfonyl)-N^2^-(2-(trifluoromethyl)acryloyl)-L-argininate Conjugate 11

To a stirred solution of **9** (125 mg, 0.19 mmol) in DCM (4.3 mL) at 0°C, DIPEA (36.5 µL, 0.21 mmol) was added followed by a solution of 2-(trifluoromethyl)acryloyl chloride **11** (33.2 mg, 0.21 mmol) in DCM (1 mL) dropwise. The solution was stirred at 0°C for one hour. The solution was diluted with a saturated aqueous solution of NH_4_Cl and extracted with DCM. The collected organic phases were dried over Na_2_SO_4_, filtered and the solvent evaporated under pressure. The crude was purified by FC affording 70 mg of **12** (0.19 mmol, 47% yield) as a white solid.

### Synthesis of PAMAM-Arg Conjugates 1–2

To a solution of the Michael acceptors **11** (n*1.5 equiv., where n = number of the outer primary amines of the dendrimer, namely 16 for PAMAM-G2 and 64 for PAMAM G4) in MeOH (0.5 mL) a solution of the PAMAM dendrimer (10 mg) in MeOH (0.5 mL) was added dropwise and the solution stirred overnight at room temperature. The solvent was evaporated, the crude dissolved in a TFA/H_2_O/TIPS (95:2.5:2.5) mixture and the solution stirred at rt for three hours. The conjugates were precipitated in diethyl ether and purified via dialysis with a SpectraPor® RC membrane (MWCO = 1 kDa) against deionized water for 2 days. The products were lyophilized obtaining PAMAM-Arg conjugates **1–2** as fluffy white solids.

### Viability Protocols

For cytotoxic tests, MTT assays were performed on human umbilical vein endothelial cells (HUVEC) treated with the different drug formulations reported in this work. The aqueous solution of fluorinated prodrug at 10 mg/mL was diluted with PBS and culture medium (Endothelial Cell Growth Medium from Promocell equipped with media supplement premixed in one vial) until a concentration of 0.025 mg/mL. Then 150 μL of media/conjugates solution was added to treat 30′000 cells in each well of a 48-well plate, previously incubated for 24 h, and let to growth in an incubator with 5% CO_2_ in air at 37°C and 96% relative humidity. After 48 h of incubation, MTT assay was performed by replacing the culture medium with the same amount of 1 × MTT + PBS/culture medium solution and incubating the cells for 4 h. After incubation, 200µL of DMSO was added to dilute the MTT-formazan formed by metabolically active cells and spectrophotometric measurement via TECAN SPARK® Multimode Microplate Reader of MTT-formazan at 570 nm was performed to quantify cell viability. Four replicates were performed for each polymer and the results are reported as average of the cell viability in each condition ± standard deviation.

### Drug Release Experiments

**Fig. 1:**
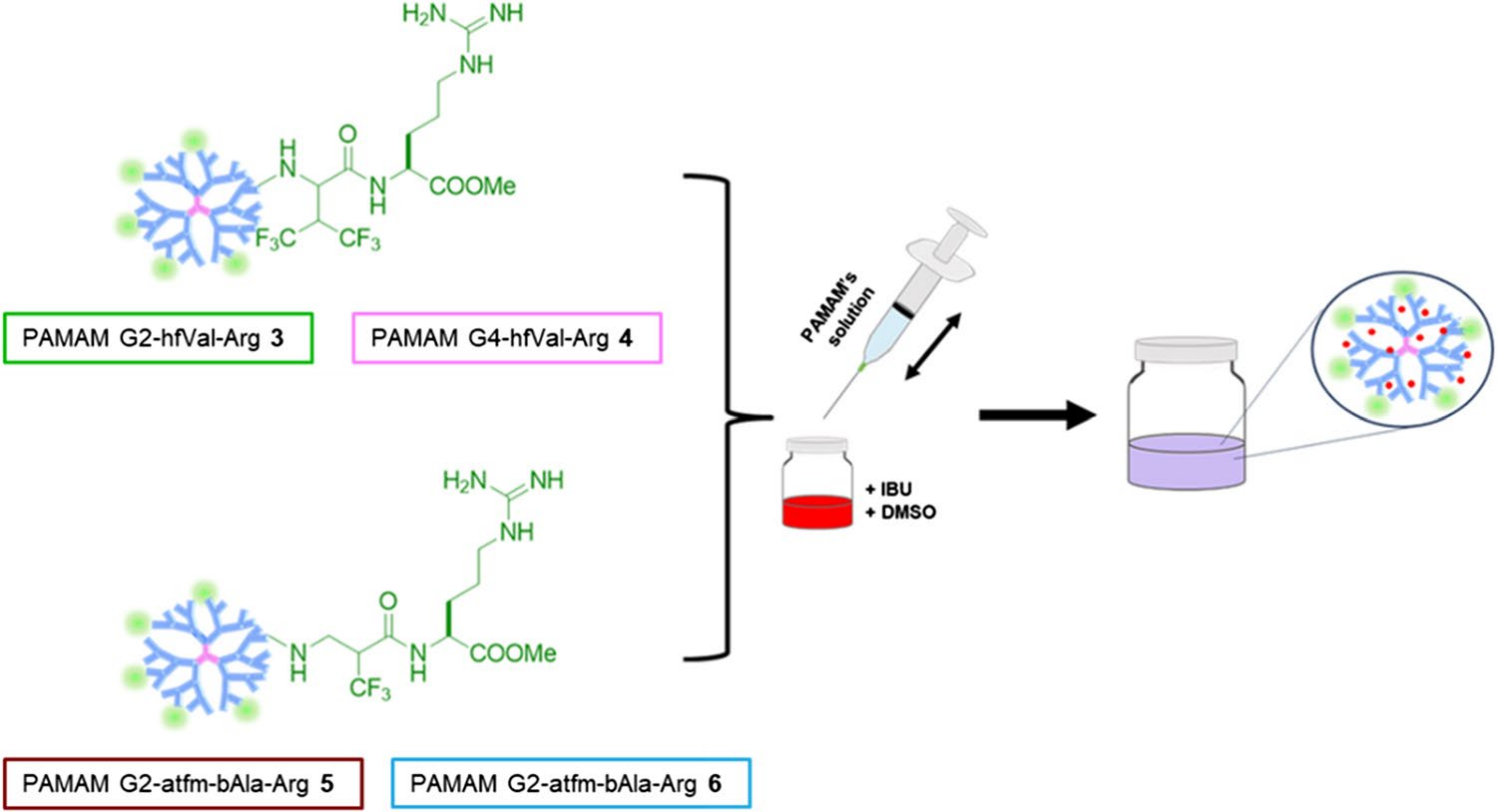
Scheme of drug encapsulation method to obtain IBU-charged fluorinated PAMAM-Arg.

For drug/dendrimer complexes preparation, a concentration of 10 mg/g (drug/polymer) was considered. As an example, 50 µg of ibuprofen were diluted in 100 µl of DMSO. Then, the drug solution was aspired by a syringe prefilled with 5 mg of dendrimers suspended in 2 mL of distilled water. The obtained mixture was ejected and aspired three times to reach an efficient drug encapsulation and filtered with a 0.45 μm nylon filter to remove non-encapsulated drug (Fig. 1).

The obtained mixture and the taken aliquot, for drug loading and release respectively, were dried under nitrogen and extracted in 1 mL of MeCN and the samples were centrifuged at 5000 rpm for 5 min, guaranteeing the precipitation of the dendrimer and the complete extraction of the drug from dendritic structure. Then, the supernatant containing the drug was analyzed via HPLC. To determine the IBU concentration from absorbance data, a calibration curve was constructed (S1) and the loading efficiency, i.e. the percentage of IBU entrapped within the dendritic structure was calculated according to Eq. (1):

1 $${\%}_{IBU loaded}=100 \times (1-\frac{{m}_{IBU,NL}}{{m}_{IBU,0}})$$

where %_IBU loaded_ is the drug loading efficiency, while m_IBU,0_ and m_IBU,NL_ are the mass of loaded IBU and the mass of IBU non- loaded, i.e. recovered in the supernatant, respectively.

The suspension of drug-loaded dendrimer was added to a dialysis membrane (SpectraPor® RC, MWCO = 1 kDa) and dialyzed against PBS for 24 h. The release profile was investigated collecting aliquots of 200 µl from the dialysis bag at fixed time points (0, 0.5 h, 1 h, 2 h, 5 h, 8 h, and 24 h) and analyzed via HPLC to quantify the residual ibuprofen according to Eq. (2):

2 $${Released}_{IBU}(t)=100 \times (1-\frac{{m}_{IBU}}{{m}_{IBU,0h}})$$

where Released_IBU_(t) is the percentage of ibuprofene released at the specific time t and m_IBU_ and m_IBU,0 h_ are the amounts of ibuprofen in the membrane at fixed time point t and time zero, respectively.

For the prodrug system, 5 mg of IBU-dendrimer conjugate were suspended in 2 mL of distilled water to be comparable to the complexes solution concentration described above. With respect to IBU/fluorinated PAMAM-Arg complexes, fluorinated prodrug dendrimers **1–2** were loaded in the same membranes (SpectraPor® RC, MWCO = 1 kDa) and dialyzed against PBS for 130 h. The release profile was investigated collecting aliquots of 7 mL outside the dialysis bag at fixed time points (0, 2 h, 5 h, 8 h, 24 h, 48 h, 52 h, 72 h, 96 h, 120 h, 130 h) and analyzed via HPLC to quantify the released ibuprofen. These aliquots were frozen in liquid nitrogen for 5 min and lyophilized on a Telstar Lyoquest freeze-drier overnight. The drug was finally extracted in ACN before quantification according to Eq. (3):

3 $${Released}_{IBU}\left({t}_{n}\right)={Released}_{IBU}\left({t}_{n-1}\right)+100 \times (\frac{{m}_{IBU}({t}_{n})}{{m}_{IBU,0h}})$$

where Released_IBU_(t_n_) is the percentage of ibuprofen released at the fixed time point t_n_, Released_IBU_(t_n−1_) is the percentage of released ibuprofen at the previous time point t_n−1_, m_IBU_(t_n_) is the mass of ibuprofen recovered at the time point t_n_ and m_IBU,0 h_ is the mass of ibuprofen at time zero, i.e. the amount of ibuprofen conjugated to the dendritic structure. After every aliquot collection, the dialysis medium was replaced with fresh one to ensure suitable sink conditions.

### HPLC Analysis

To evaluate the IBU concentration in each sample taken during the release experiments, high performance liquid chromatography (HPLC) was performed on a Jasco 2000 Series chromatography equipped with a variable wavelength detector and a reversed-phase C18 column (Restek, 250 mm in length, 4.6 mm internal diameter, and 5 μm particle size), maintaining the detection at 240 nm. The analyses were performed at 1 mL/min in isocratic conditions with a mobile phase of 60/40 vol/vol ACN/20 mM phosphate buffer at pH 6. The ibuprofen concentration was evaluated from a calibration curve correlating the peak area (mAU * min) to the known concentration from the analysis of 7 samples, obtaining a correlation coefficient of the linear regression of the experimental data equal to 0.99 (Figure S1).

### Statistical Analysis

All data were analyzed statistically using Origin software. For both HPLC analysis and *in vitro* tests, 4 independent experiments were conducted for each polymer. All the result were expressed as the mean ± standard deviations. Student’s t-test was used to determine statistical significance.

## Results and Discussion

### Synthesis of Fluorinated IBU-PAMAM-Arg Conjugates

**Scheme 1:**
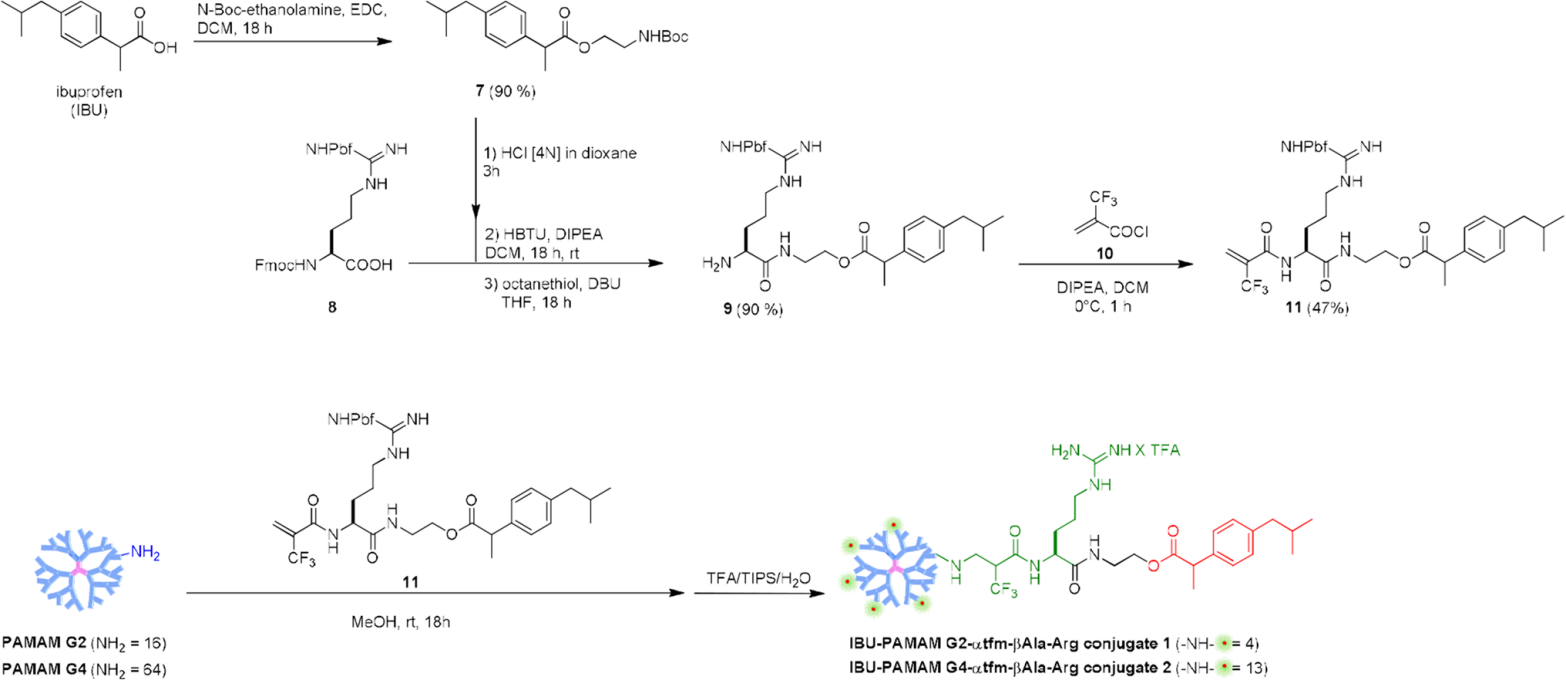
Synthesis of fluorinated IBU-conjugated Arg-Michael acceptor **11** and of IBU fluorinated PAMAM-Arg conjugates **1**–**2**.

In the past years, our research group has developed a simple approach to include a fluorinated component into organic molecules/polymers exploiting Michael addition reaction of nucleophiles to α,β-unsaturated carbon–carbon double bonds that possess a highly electronegative trifluoromethyl group (tfm) in α or β position [45–48]. This strategy enhances the reactivity of the electrophilic alkene and has been utilized for the synthesis of peptidomimetics, both in solution and solid phase. The favorable chemical features of this reaction, such as the unnecessary use of coupling reagents, the high reaction rate in mild conditions, and the lack of by-product formation allowed us lately to exploit the Michael addition reaction to obtain an efficient “click” functionalization of the terminal primary amino groups of PAMAM dendrimers with ad hoc developed fluorinated Michael acceptors. Basing on our previous work [15], where fluorinated PAMAM-Arg conjugates **3–6** were synthesized via a “click” functionalization with two Michael acceptors containing both guanidino functional groups and one or two tfm groups, we have synthesized novel fluorinated IBU-Arg-containing Michael acceptors **11** having only one tfm and a guanidino function (Scheme 1), both important to enhance the cellular internalization of the final PAMAM prodrugs and to maintain high cellular viability. The decision to opt for a linker with one tfm group instead of two (as in conjugates **3–4**) arose from the fact that, being the tfm group in α position of the unsaturated carbon–carbon double bond, the corresponding linker is much more reactive in the click functionalization with the PAMAM dendrimers allowing us to get higher yields in milder conditions (i.e. functionalization carried at room temperature). Furthermore, the presence of a single tfm group would lead to a single signal in the ^19^F NMR spectra of the final conjugates, which is advantageous for molecular fluorine tracking and imaging purposes through ^19^F-MRI [49]. Specifically, IBU was coupled with N-Boc-ethanolamine leading to compound **7** possessing an ester functional group, whose hydrolysis could be exploited for the release of the drug (Scheme 1). After Boc-removal triggered by treating **7** with HCl, the forming free amine was coupled with Fmoc-Arg(Pbf)-OH **8** yielding, after Fmoc-deprotection, IBU-arginine conjugate **9** which was acylated with α-(trifluoromethyl)acryloyl chloride **10** [45] producing the final Michael acceptor **11** in good yields.

PAMAM G2 and PAMAM G4 dendrimers were clicked with compound **11** following the same reaction condition used for the synthesis of conjugates **5–6**, namely in MeOH at rt for 18 h. After deprotection of the guanidino group with a mixture of TFA/TIPS/H_2_O, dialysis and lyophilization, derivatives **1–2** were obtained. According to our previous studies [14–16], the functionalization degrees (FDs), defined as the number of available amines on the surface of PAMAM effectively functionalized with IBU, were measured by ^1^H NMR, through the integration of signals belonging to the PAMAM dendrimer *versus* signals belonging to the IBU-Arg-containing arm. In Fig. 1A, the ^1^H NMR spectra of undecorated PAMAM G4 (blue spectrum), model compound **12** prepared by addition of N-(2-aminoethyl)acetamide with **10** (green spectrum), and IBU-PAMAM G4-α-tfm-β-Ala-Arg **2** (red spectrum), all recorded in D_2_O, are represented as an example.

**Fig. 2:**
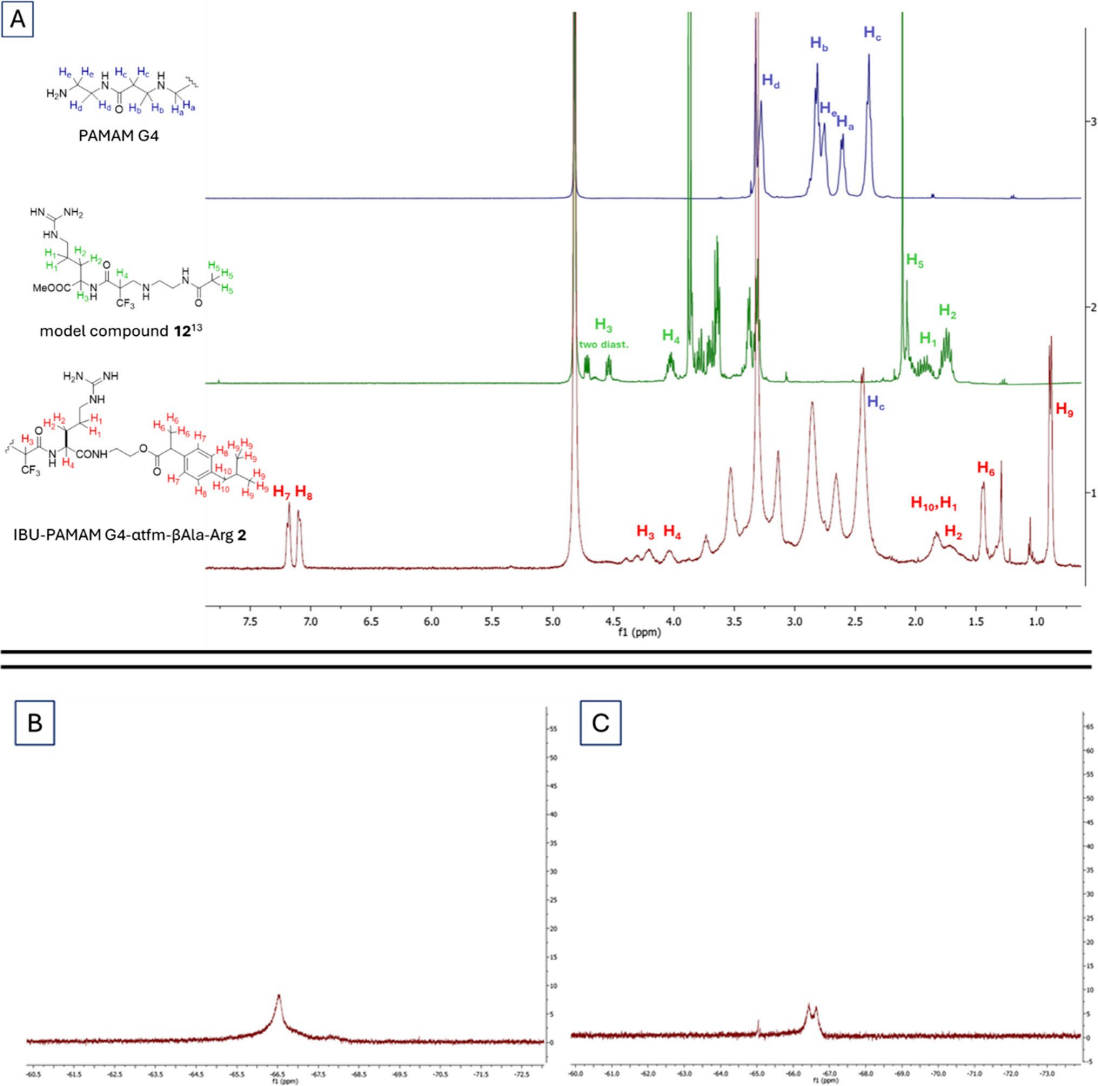
In (**A**) ^1^H NMR spectra recorded in D_2_O of PAMAM G4 (blue spectrum), model compound **12** (green spectrum), and IBU-PAMAM G4-αtfm-βAla-Arg **2** (red spectrum). In (**B**) and (**C**) are reported the fluorine signal of **1** and **2**, respectively, at -66.5 ppm.

The characteristic protons belonging to the decorating arm distinctly visible in the spectrum of IBU-PAMAM G4-α-tfm-β-Ala-Arg **2** (red spectrum, Fig. 2A) and absent in the spectrum of undecorated PAMAM G4 (blue spectrum, Fig. 2A) can be grouped in four sets of signals: 1) the six hydrogens H_9_ belonging to the two methyl groups of the isobutyl substituent of IBU which resonate around 0.8 ppm; 2) the three protons H_6_ belonging to the other methyl group of IBU resonating at 1.4 ppm; 3) the broad signal between 1.6 and 1.8 ppm due to the resonance of hydrogens H_1_ and H_2_ (four hydrogen in total) of the arginine side chain and of the two protons H_10_ of the methylene moiety of the IBU isobutyl group, and 4) the four aromatic hydrogens resonating around 7.2 ppm. These sets of protons can be integrated *versus* the protons Hc belonging to the methylene group in α position to the carbonyl groups in PAMAM dendrimers (56 for PAMAM G2 and 248 for PAMAM G4) resonating between 2.6 and 2.8 ppm (see Supplementary materials). In addition, an intense fluorine signal from the dendrimers could be measured via ^19^F NMR (Fig. 2B and C), testifying the possible implementation of these prodrugs as theragnostic tools, combining therapy and diagnosis via fluorine MRI.

The chemical characterization, including the chemical shift of the ^19^F NMR signals and the calculated molecular weights (MWs) of the two fluorinated prodrug dendrimers synthesized are collected in Table I. Based on this analysis, the FDs for conjugates **1** and **2** were 28.1% and 23.4%, respectively. In contrast to **3–4**, which exhibited FDs of 65.6% and 66.5%, respectively,^15^ the reduced FDs observed with the IBU-fluorinated Michael acceptor **11** are likely attributed to the increased steric hindrance of the resulting conjugate. Notably, a similar steric hindrance issue was encountered with **3–4**, produced through click-functionalization with bis-trifluoromethyl Michael acceptors.

**Table I:**
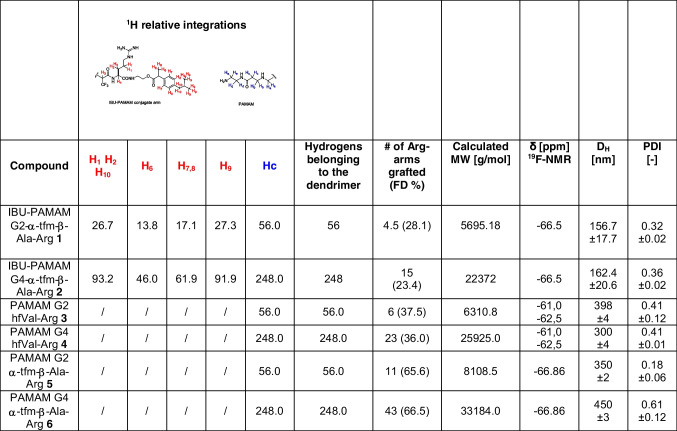
Chemical Characterization and Intensity-average Hydrodynamic Diameter (D_H_) and Polydispersity Index (PDI) of Fluorinated IBU-PAMAM-Arg Conjugates **1–2** and PAMAM-Arg Conjugates **3–6**.^15^

The hydrodynamic diameter (D_H_) and polydispersity index (PDI) of these fluorinated IBU-PAMAM-Arg conjugates **1**–**2** was investigated by dynamic light scattering (DLS). Interestingly, as shown in Table I, IBU-conjugation lead to the formation of nanoparticles in buffer solutions at 37°C smaller than those obtained with fluorinated PAMAM-Arg **3–6**, generating nanoparticles with D_H_ of 157 and 162 nm for **1** and **2**, respectively, compatible with their systemic administration.

### Drug Loading Efficiency and Drug Release Study from IBU/PAMAM Complexes

**Fig. 3:**
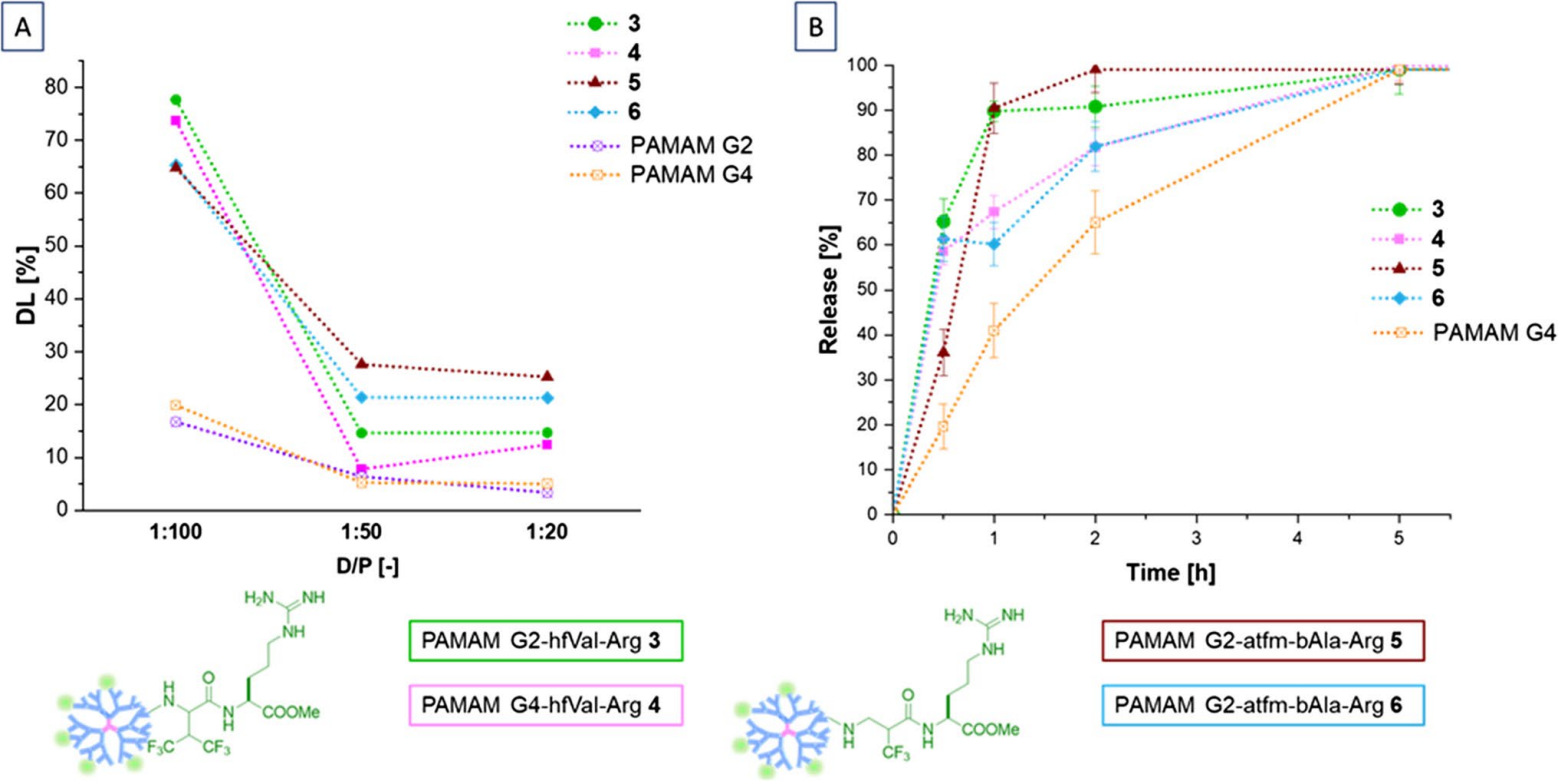
Scheme of drug encapsulation method**,** drug loading efficiency, and drug release profiles performed at 37°C and at physiologic pH of IBU-charged fluorinated PAMAM-Arg; (**A**) report the drug loading efficiency of undecorated PAMAM and fluorinated PAMAM, respectively, showing the higher drug loading of decorated dendrimers. In (**B**), the IBU release profile at physiologic pH is reported for the different carriers compared to undecorated PAMAM G4.

First, we explored the capability of the four fluorinated PAMAM dendrimers **3–6** to physically encapsulate and release IBU in physiological conditions. Drug loading (DL) was performed following a simple nanoprecipitation strategy, reported in literature for an efficient encapsulation of poorly water-soluble drugs, using a syringe and a needle as mixing chambers without the need of postprocessing steps (Fig. 3A) [44]. The drug loading efficiencies at three different drug/polymer ratios (D/P) were evaluated at physiological condition (Fig. 3B and C). Interestingly, for all the considered D/P ratios we observed a significantly higher drug loading efficiency using the fluorinated PAMAM conjugates **3–6** than the undecorated dendrimers, showing the pivotal role of the functional groups, i.e. the hydrophobic tfm group and the guanidino moiety, in stimulating drug encapsulation. As expected, all the dendrimers showed a DL that increases when reducing the D/P ratio. Furthermore, all the obtained conjugates **3–6** seem to maintain a higher DL also at low D/P compared to undecorated PAMAM G4, probably thank to a compacted structure, rising in a coupled positive charge and fluorine density distribution along the surface, thus giving a major electrostatic and hydrophobic interaction with the drug, respectively.

On the other hand, for all the fluorinated PAMAMs **3–6** a fast release is observed within five hours, with no significant differences between fluorinated PAMAMs with different degrees of fluorination and/or guanidino groups content (Fig. 3B and S1 in Supplementary information). It appears that fluorinated PAMAM G4 derivatives exhibit a slightly slower release during the initial two hours compared to fluorinated PAMAM G2 derivatives. Nevertheless, there is an observable increase in the release rate, leading to a release profile similar to that of fluorinated PAMAM G2 over time. The observed fast release is probably the result of weak electrostatic and hydrophobic interactions between IBU and the surface positive charges on guanidino groups and the fluorine moieties, respectively. Moreover, the steric hindrance introduced by the arginine-derived building block structure may contribute to the limited entry of the drug into the dendritic core, influencing the overall release behavior. Therefore, alternative strategies must be implemented to prolong the release of the loaded drug for therapy.

**Fig. 4:**
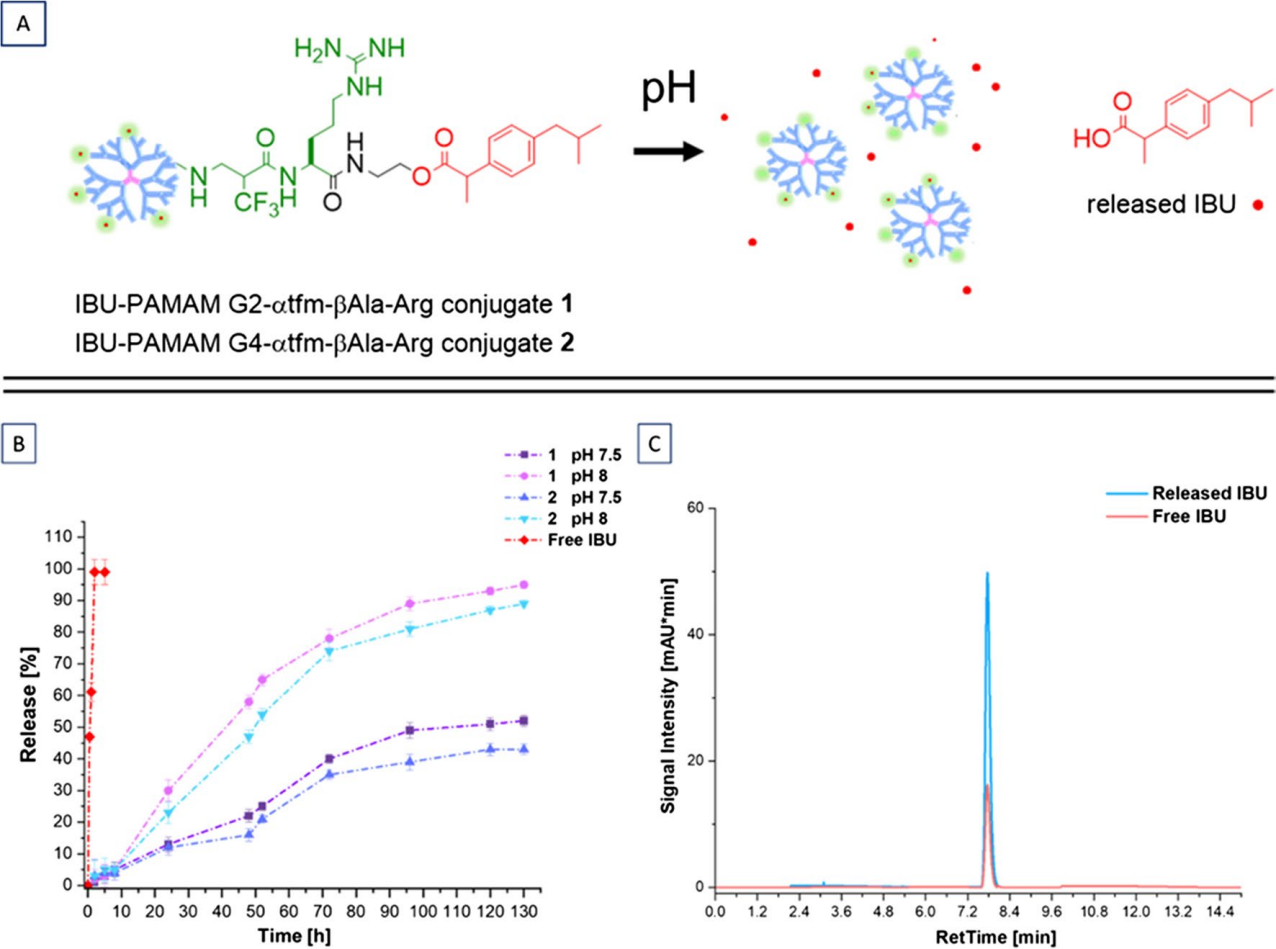
(**A**) schematic representation of IBU release. (**B**) drug release profiles performed at 37°C and at physiologic pH and pH = 8 from fluorinated IBU-PAMAM-Arg conjugates in comparison to free IBU release at physiologic pH. (**C**) HPLC chromatogram comparing IBU released from the carriers and IBU standard, showing the same retention time at 7.76 min.

For this reason, novel fluorinated IBU-PAMAM-Arg conjugates **1–2** acting as esterase-responsive prodrugs were synthesized, following the “click” functionalization shown above. These systems were obtained in high yield and good FDs, guaranteeing the presence of suitable amount of covalently linked drug that can undergo a controlled release due to the hydrolysis of labile ester bonds in both physiological conditions and pH = 8 (Fig. 4).

The drug release investigation was performed at both physiological pH (pH = 7.5) and alkaline pH (pH = 8), the latter mimicking a higher concentration of esterase. As depicted in Fig. 3A a remarkable and promising controlled release is showed for both fluorinated prodrugs **1** and **2**, without significant differences from different PAMAM generations, which is not surprising considering that in both conjugates IBU is linked to the outside of the dendrimers and that its release depends on a chemical hydrolysis of the ester linkage rather than on weaker intermolecular forces which are affected by the size of the dendrimer. In contrast to the quick release of both IBU/PAMAM complexes **3**–**6**, IBU/undecorated G4 complex, and free IBU for both pH conditions, a sustained IBU release could be preserved in this case for more than 5 days. Moreover, as we expected, a higher release at alkaline pH = 8, which mimics the esterase action inside the human body, can be observed with respect to physiological pH condition, as a consequence of an accelerated ester bond hydrolysis, as reported for other prodrugs [50, 51]. Specifically, an almost complete release of IBU (close to 80%) was achieved for both **1** and **2** at pH = 8 after 72 h in contact with alkaline PBS, while the maximum amount of released IBU observed at physiological pH is near to 50% after the same time.

The results obtained confirm the pH-responsive behavior of the synthesized fluorinated PAMAM prodrugs and its efficacy in ensuring a sustained release for IBU. This underscores the efficacy of the "click" functionalization strategy developed, showcasing its versatility not only in generating promising fluorinated non-viral vectors for gene therapy as previously demonstrated [15] but also in synthesizing high-performance fluorinated prodrugs.

### *In Vitro* Cytotoxicity for Fluorinated IBU-PAMAM-Arg Conjugates

**Fig. 5:**
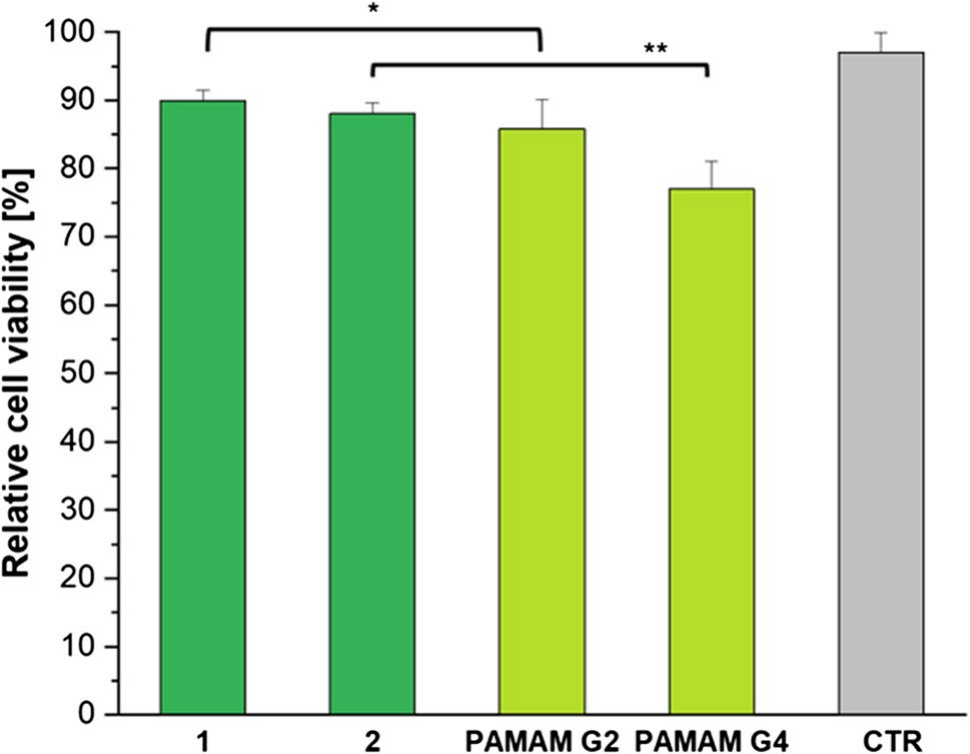
Cytotoxicity of fluorinated IBU-PAMAM-Arg conjugates **1–2** after 48 h. For negative control (CTR), HUVECs without prodrug vehicles were considered. Error bars are standard deviation and statistical significance is **p < 0.01 for prodrug 2 compared to undecorated PAMAM G4 and ns: *p < 0.06 for prodrug 1 compared to undecorated PAMAM G2.

The safety of the drug delivery systems is a fundamental criterion for clinical application. For this reason, the cytotoxicity of IBU-PAMAM-Arg conjugates **1–2** was evaluated (Fig. 5).

HUVECs were used as a model primary cell system for cytotoxicity evaluation of the dendrimers and for a comparative study with undecorated PAMAM and fluorinated-PAMAM-Arg conjugate **3**–**4** [15]. As depicted in Fig. 4, both fluorinated PAMAM prodrugs showed low cytotoxicity after 48 h, with a cellular viability comparable to that obtained for the control group, corresponding to untreated cells. By comparison with the data taken after 24 h (Figure S3 in Supplementary Information), no significant changes in cell viability were observed, which leads us to assume that no temporal effects are observable with these dendrimers. The obtained results confirm that the covalent binding of IBU to PAMAM did not have an impact on the cell viability compared to the non-functionalized dendrimers. Indeed, non-significant difference in viable cell density was observed for compound 1 and PAMAM G2, while compound 2 actually reduced the cytotoxicity compared to the undecorated PAMAM G4 (*p* < 0.001), underlining the safety of the prodrugs developed in this work and their potential use in drug delivery [50].

## Conclusion

The achievement of sustained drug release for prolonged time, activated on demand, is the principal aim for an efficient drug delivery system. Drug encapsulation exploiting non-covalent interactions between drug and polymeric vehicles is an established strategy, but several issues in specific drug targeting, controlled release, and drug/vehicles complex stability remain, often reflected in an initial and unpredictable burst release. In this context, carrier prodrugs emerge as a promising alternative for enhancing drug delivery. This entails exploring innovative synthetic strategies to create polymeric vehicles that can be finely tuned as versatile devices, offering synergistic biomedical applications.

Herein, we reported the design and synthesis of a pH-responsive fluorinated IBU-Arg Michael acceptor, and its use in decorating part of the outer free amines of PAMAM G2 and G4 dendrimers. A comparative study on IBU release was conducted considering physical drug encapsulation with fluorinated PAMAM-Arg G2 and G4 **3**–**6** and the new esterase responsive IBU-PAMAM-Arg **1**–**2** acting as prodrugs. The dendrimers **3**–**6** demonstrated a higher encapsulation efficiency compared to undecorated PAMAM G2 and G4, but fast release rate at physiological pH. Conversely, both conjugates **1** and **2** exhibited high drug loading as well as the possibility of sustaining its release for more than 5 days. This could be enhanced by increasing the pH to 8, to mimic a higher concentration of esterase, demonstrating the on-demand drug release achievable with these vectors.

Furthermore, both the synthesized systems **1** and **2** presented very low cytotoxicity, that coupled to great stability, controlled and pH-dependent release behavior, gathers promising features as multifunctional drug delivery vehicles with a possible implementation in targeted molecular imaging and diagnostic ^19^F-MRI.

### Supplementary Information

Below is the link to the electronic supplementary material.

Supplementary file1 (PDF 908 KB)

## Data Availability

The original contributions presented in the study are included in the article and Supplementary Information; further inquiries can be directed to the corresponding authors.
