# Supplementary material for: Fluorinated PAMAM-Arginine Carrier Prodrugs for pH-Sensitive Sustained Ibuprofen Delivery
Source: Pharm Res. 2024 Jul 24;41(8):1725–36. doi: 10.1007/s11095-024-03747-6 (PMC11362194; doi:10.1007/s11095-024-03747-6)

SUPPLEMENTARY INFORMATION

Fluorinated PAMAM-Arginine Carrier Prodrugs for pH-Sensitive Sustained Ibuprofen Delivery

Carola Romani,[§] Mattia Sponchioni,[§,*] Alessandro Volonterio[§,†,*]

Department of Chemistry, Materials and Chemical Engineering "Giulio Natta", Politecnico di Milano, via Mancinelli 7, 20131 Milano, Italy; Consiglio Nazionale delle Ricerche, Istituto di Scienze e Tecnologie Chimiche "Giulio Natta" (SCITEC), Via Mario Bianco 9, 20131 Milan, Italy.

Table of contents

Page S2	Chemical characterization of all new compounds.
Pages S3-S8	Copies of the ^{1}H NMR, ^{19}F NMR, ^{13}C NMR and ESI MS spectra of all new compounds
Pages S9-S10	Copies of the ^{1}H NMR and ^{19}F NMR of IBU-PAMAM-Arg conjugates **1-2**
Page S11	Figure S1 showing the IBU release profile at physiologic pH for the different carriers compared to undecorated PAMAM G4 in 24 hours.
Page S12	Figure S2 of Ibuprofen calibration curve for HPLC analyses.
Page S13	Figure S3 showing the cytotoxicity of fluorinated PAMAM-Arg conjugates **3-6** after 24h.

2-((tert-butoxycarbonyl)amino)ethyl 2-(4-isobutylphenyl)propanoate, 7. ^{1}H NMR (400 MHz, $CDCl_3$) δ 7.19 (d, J = 8.1 Hz, 2H), 7.10 (d, J = 8.1 Hz, 2H), 4.53 (s, 1H), 4.18 – 4.05 (m, 2H), 3.70 (d, J = 7.2 Hz, 1H), 3.30 (s, 2H), 2.45 (d, J = 7.2 Hz, 2H), 1.85 (dt, J = 13.5, 6.8 Hz, 1H), 1.49 (d, J = 7.2 Hz, 3H), 1.43 (s, 9H), 0.90 (d, J = 6.6 Hz, 6H).; ^{13}C NMR (101 MHz, $CDCl_3$) δ 175.0, 168.7, 141.1, 138.1, 129.9, 127.5, 80.0, 64.2, 45.53, 45.49, 40.1, 30.6, 28.8, 22.8, 18.8; ESI m/z 372.2 $[M+Na, (64)]^+$, $[M+K, (100)]^+$; Anal. calcd. for $C_{20}H_{31}NO_4$: C, 68.74; H, 8.94; N, 4.01; found: C 68.75, H 8.93, N 4.01.

IBU-N$^{\omega}$-((2,2,4,6,7-pentamethyl-2,3-dihydrobenzofuran-5-yl)sulfonyl)-N^{2}-L-argininate-NH_2, 9. ^{1}H NMR (400 MHz, CD_3OD) δ 7.16 (d, *J* = 8.0 Hz, 2H), 7.06 (d, *J* = 7.8 Hz, 2H), 4.22 – 4.13 (m, 1H), 4.10 – 4.00 (m, 1H), 3.70 (t, *J* = 7.1 Hz, 1H), 3.48 – 3.32 (m, 2H), 3.22 (dt, *J* = 5.9, 2.9 Hz, 1H), 3.12 (s, 2H), 2.97 (s, 2H), 2.57 (s, 3H), 2.51 (s, 3H), 2.41 (d, *J* = 7.2 Hz, 2H), 2.06 (s, 3H), 1.86 – 1.77 (m, 1H), 1.59 (ddd, *J* = 14.6, 8.0, 5.3 Hz, 1H), 1.52 – 1.44 (m, 3H), 1.44 – 1.40 (m, 9H), 0.87 (d, *J* = 6.6 Hz, 6H).; ^{13}C NMR (101 MHz, CD_3OD) δ 177.6, 176.3, 169.1, 159.8, 141.7, 139.3, 134.5, 133.5, 130.4, 128.3, 126.0, 118.4, 107.9, 87.6, 64.1, 55.6, 46.2, 46.0, 43.9, 39.3, 33.3, 31.4, 28.7, 22.7, 19.6, 19.1, 18.4, 12.5; ESI m/z 658.4 $[M+H, (100)]^+$; Anal. calcd. for $C_{34}H_{51}N_5O_6S$: C, 62.07; H, 7.81; N, 10.65; found: C, 62.09; H, 7.80; N, 10.67.

IBU-N$^{\omega}$-((2,2,4,6,7-pentamethyl-2,3-dihydrobenzofuran-5-yl)sulfonyl)-N^{2}-(2-(trifluoromethyl)acryloyl)-L-argininate, 11. ^{1}H NMR (400 MHz, $CDCl_3$) δ 7.12 (dt, J = 5.7, 2.8 Hz, 2H), 7.02 (d, J = 7.2 Hz, 2H), 6.38 (s, 1H), 6.16 (s, 1H), 4.61 (s, 1H), 4.24 – 4.09 (m, 1H), 4.08 – 4.02 (m, 1H), 3.71 – 3.57 (m, 1H), 3.55 – 3.36 (m, 2H), 3.24 (d, J = 5.5 Hz, 2H), 2.94 (s, 2H), 2.56 (s, 3H), 2.48 (s, 3H), 2.40 (t, J = 6.4 Hz, 2H), 2.06 (d, J = 16.3 Hz, 3H), 1.84 (m, 2H), 1.69 (m, 1H), 1.57 – 1.48 (m, 2H), 1.44 (d, J = 5.7 Hz, 6H), 1.41 (dd, J = 7.2, 1.4 Hz, 3H), 0.87 (t, J = 5.4 Hz, 6H). ^{19}F NMR ($CDCl_3$, 376 MHz) δ – 64.41 (s, 3F); ^{13}C NMR (101 MHz, $CDCl_3$) δ 174.9, 171.1, 161.6, 159.0, 156.4, 140.6, 138.4, 137.6, 132.5, 132.3, 129.3, 127.2, 124.8, 117.7, 86.5, 63.0, 60.4, 53.4, 52.6, 45.0, 43.2, 30.1, 28.6, 28.3, 28.1, 22.4, 21.0, 19.3, 18.4, 17.9, 14.2, 12.4, the CF_3 signal and the signal of the Cα to the CF_3 group were obscured due to their low intensity; ESI m/z 780.3 $[M+H, (45)]^+$, 802.3 $[M+Na, (100)]^+$, 818.2 $[M+K, (39)]^+$; Anal. calcd. for $C_{38}H_{52}F_3N_5O_7S$: C 58.52, H 6.72, N 8.98; found: C 58.50, H 6.71, N 8.97.

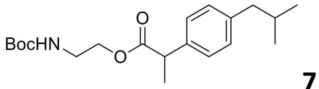

7

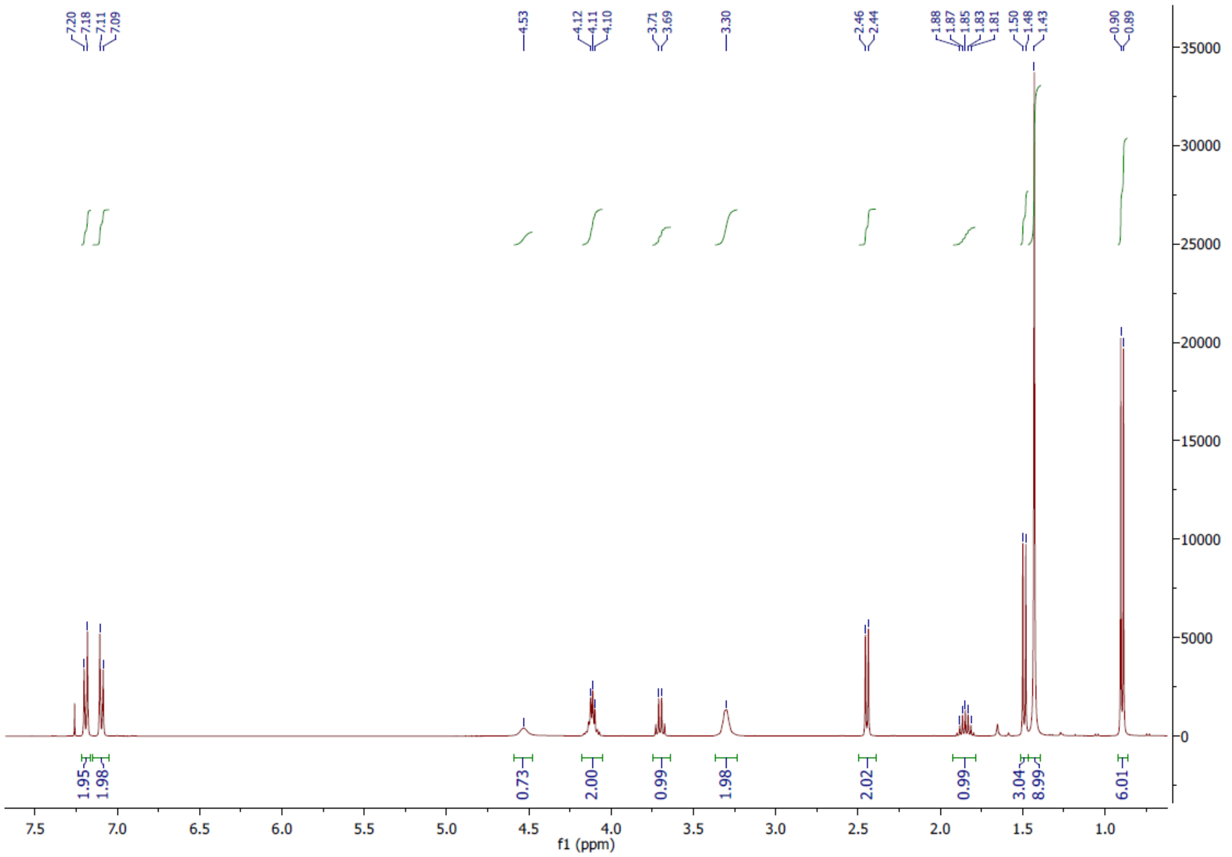

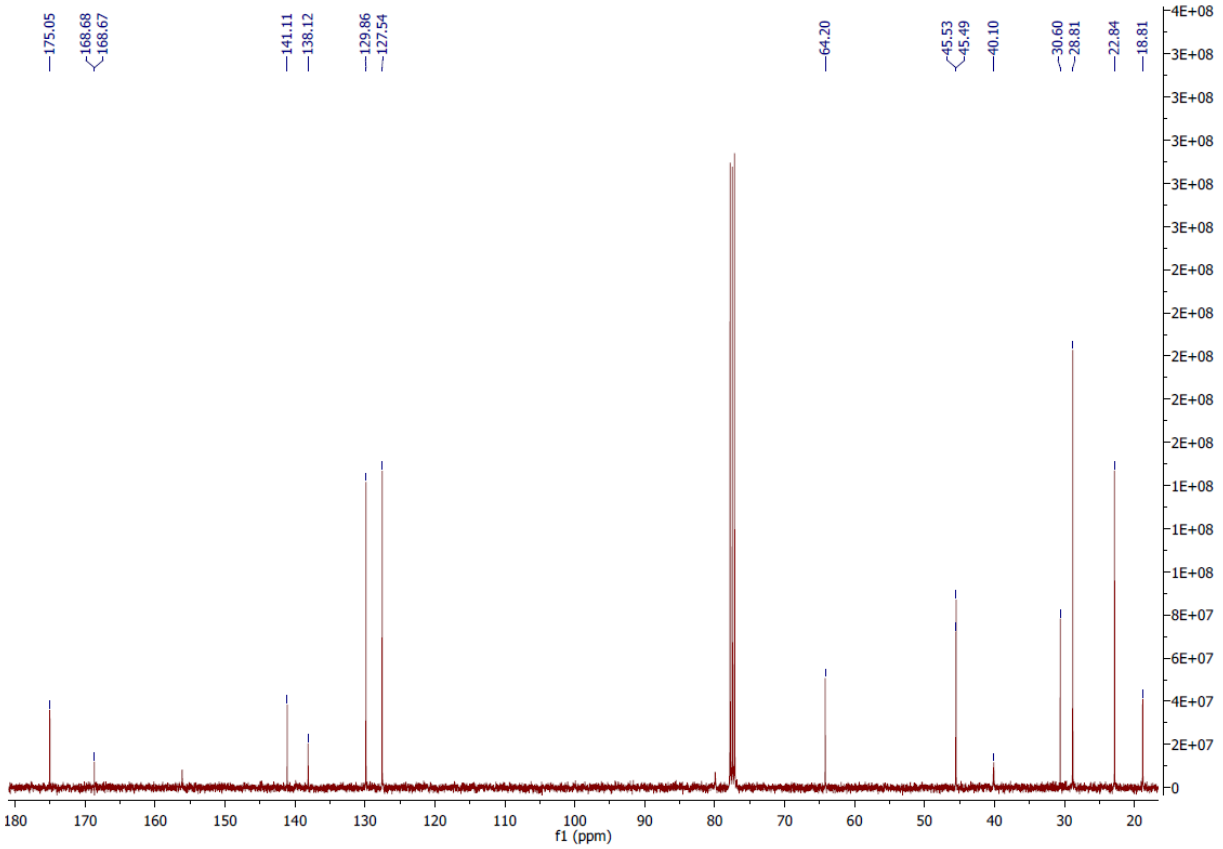

Analysis Info

Analysis Name	av cr_107.d	Acquisition Date	07/18/23 18:37:33	Operator	Gabriella
Sample Name		Method	Copy of $opt0622.MS	Instrument	esquire3000plus
Comment	1/mg/mL MeOH dil 1/100 in MeOH<br>Richiedente: Carola Romani				

Acquisition Parameter

Ion Source Type	ESI	Mass Range Mode	Std/Normal	Ion Polarity	Positive	Alternating Ion Polarity	off
Scan Begin	50 m/z	Scan End	1000 m/z	Averages	5 Spectra	Accumulation Time	33 µs
Capillary Exit	117.3 Volt	Skim 1	40.0 Volt	Trap Drive	47.0	Auto MS/MS	off

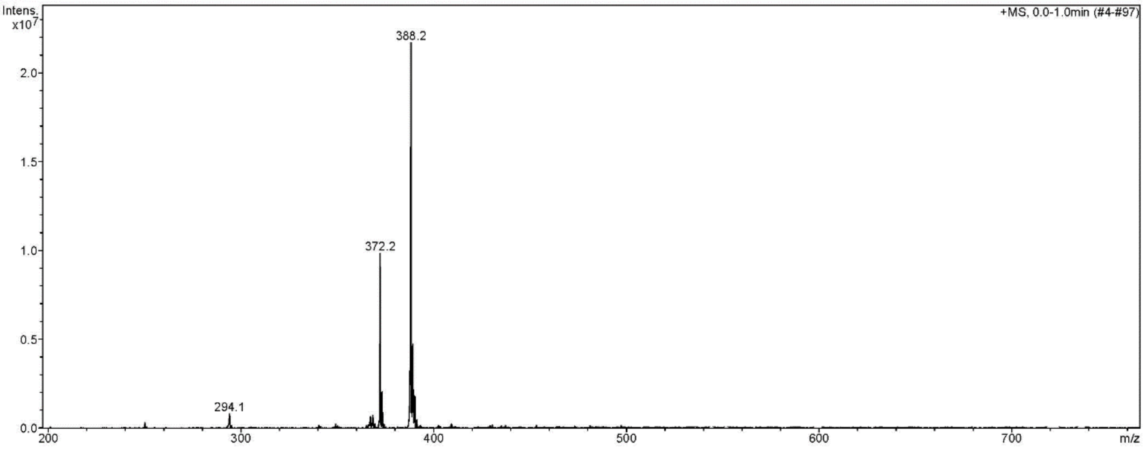

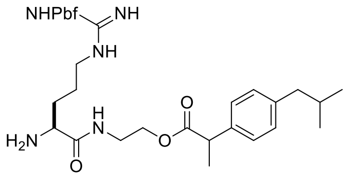

9

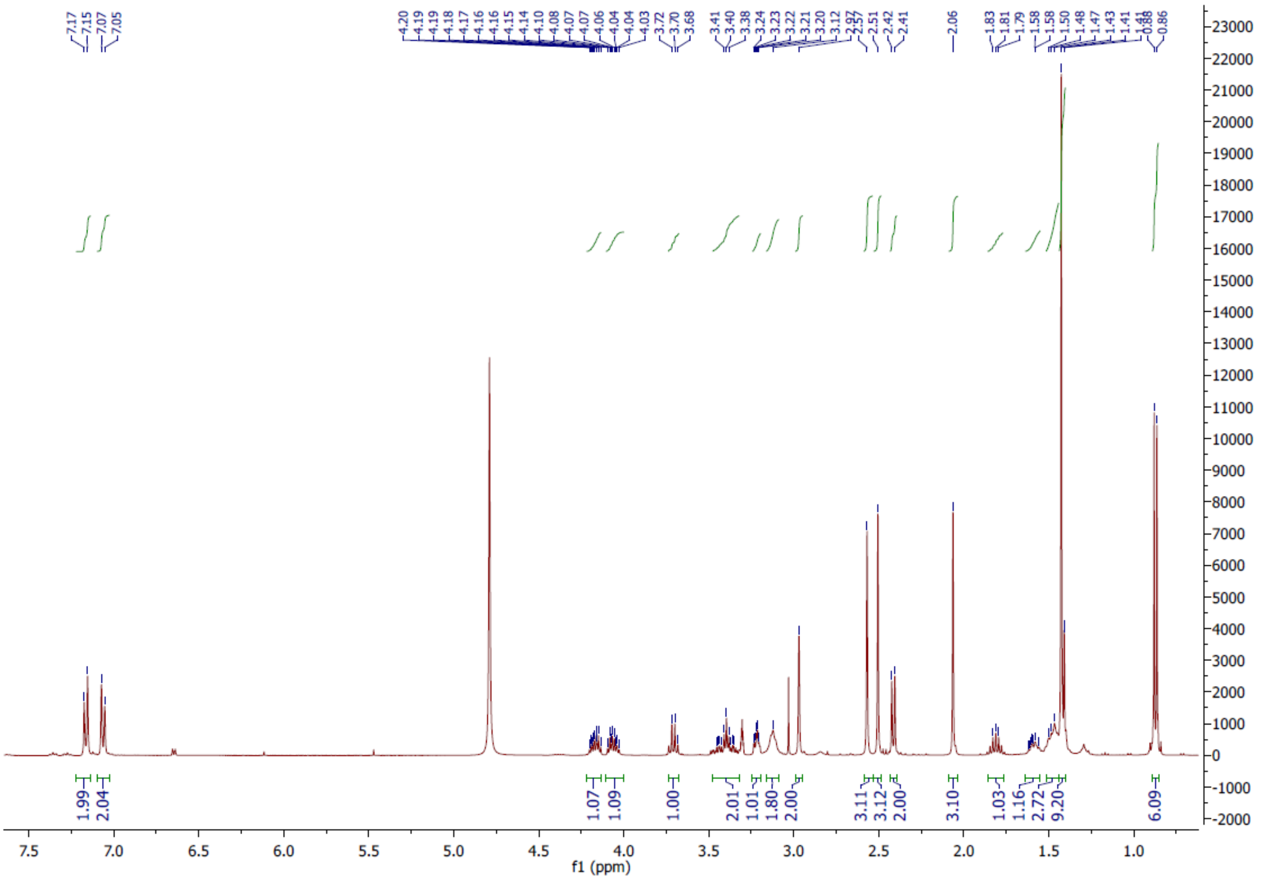

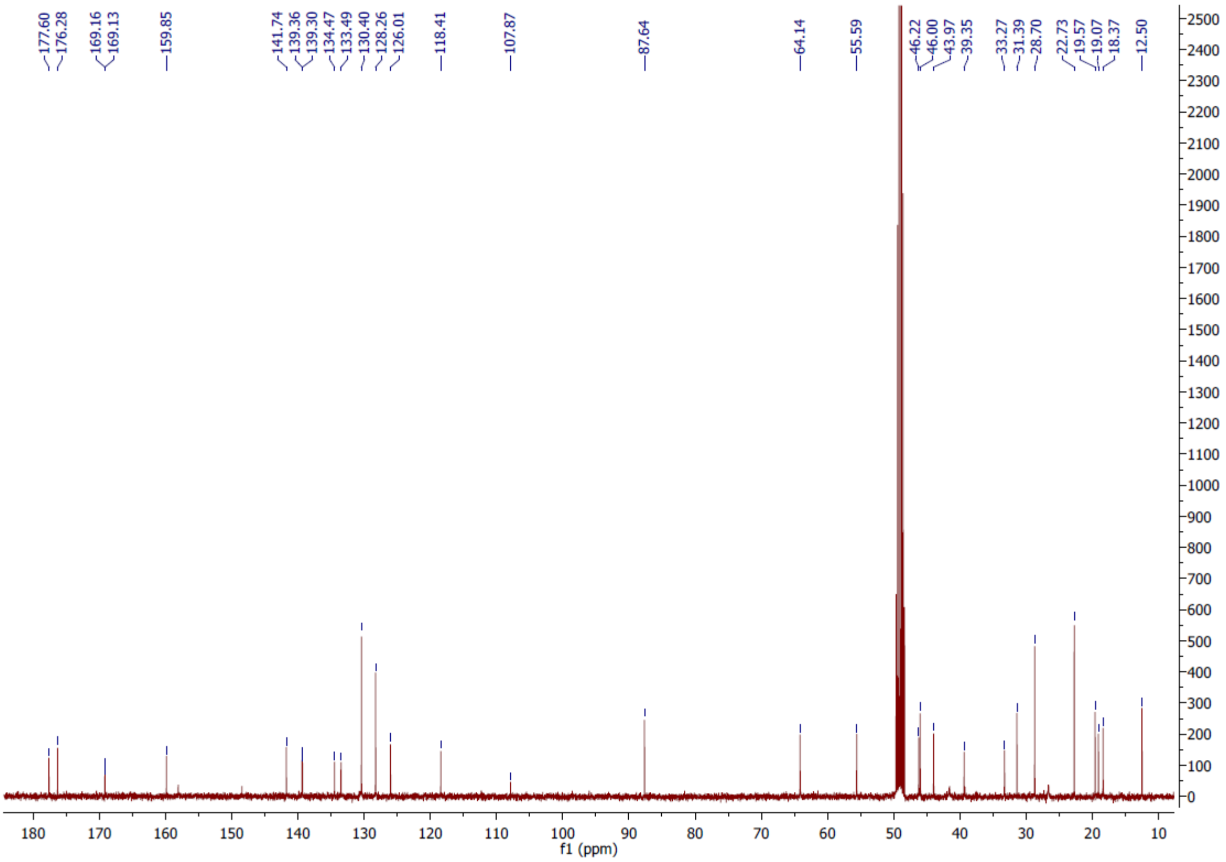

-L.G.S. - Laboratorio Grandi Strumenti - Display Report

Analysis Name	av crc_04.d	Acquisition Date	02/16/23 13:26:05	Operator	Administrator
Sample Name		Method	Copy of $opt0622.MS	Instrument	esquire3000plus
Comment	1 mg/mL dil 1:100 MeOH<br>Richiedente: Carolina Corti				

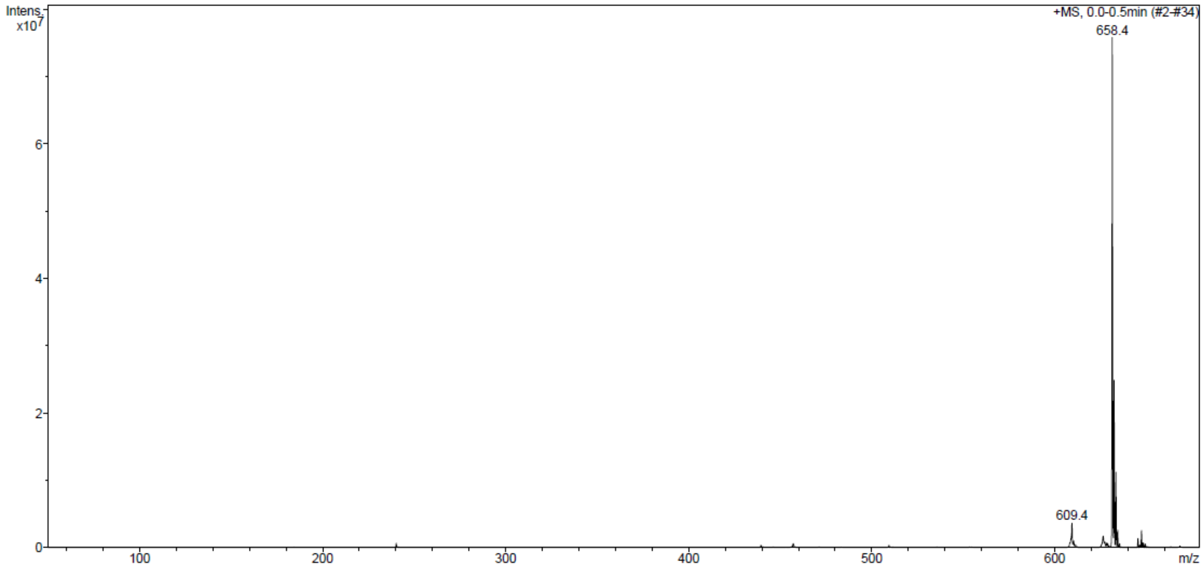

Bruker Daltonics DataAnalysis 3.1 printed: 02/16/23 13:27:12 Page 1 of 1

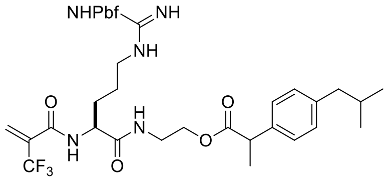
NHPbf
NH
NH
O
H
N
O
H
N
O
O
CF3
O

11

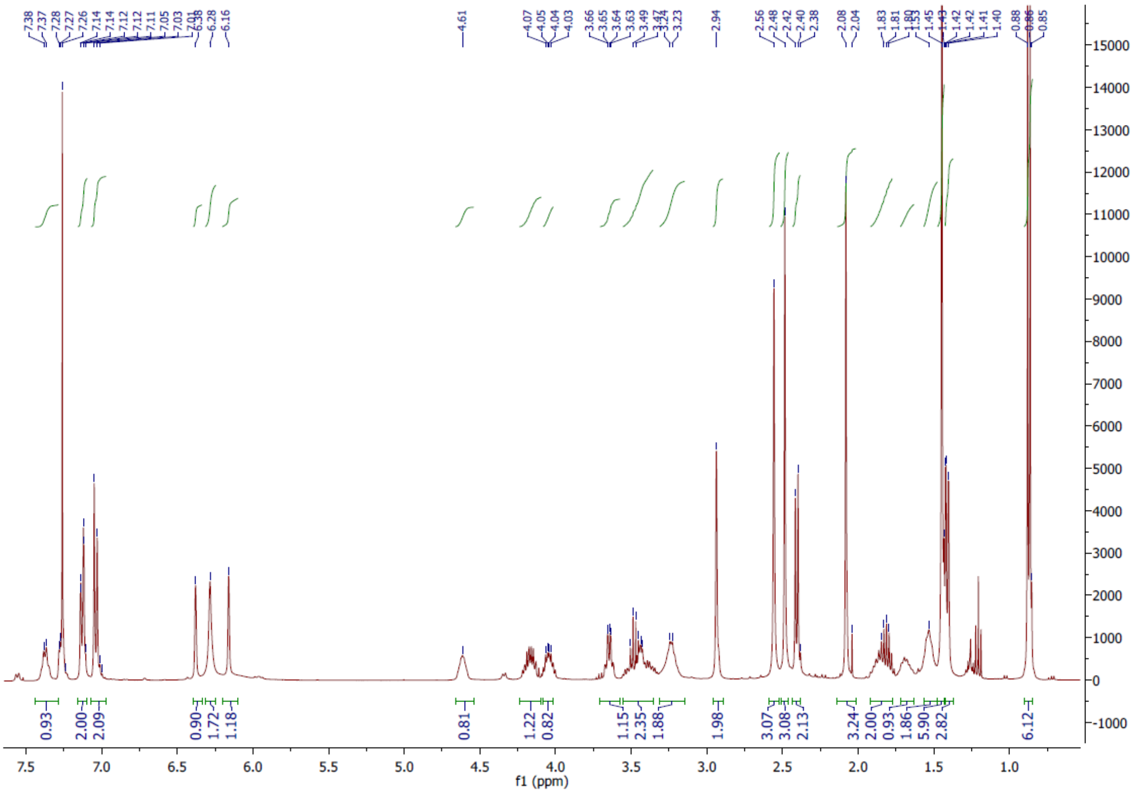
f1 (ppm)
0.93
2.00
2.09
0.90
1.72
1.18
0.81
1.22
0.82
1.15
2.35
1.88
1.98
3.07
3.08
2.13
3.24
2.00
0.93
1.86
5.90
2.82
6.12

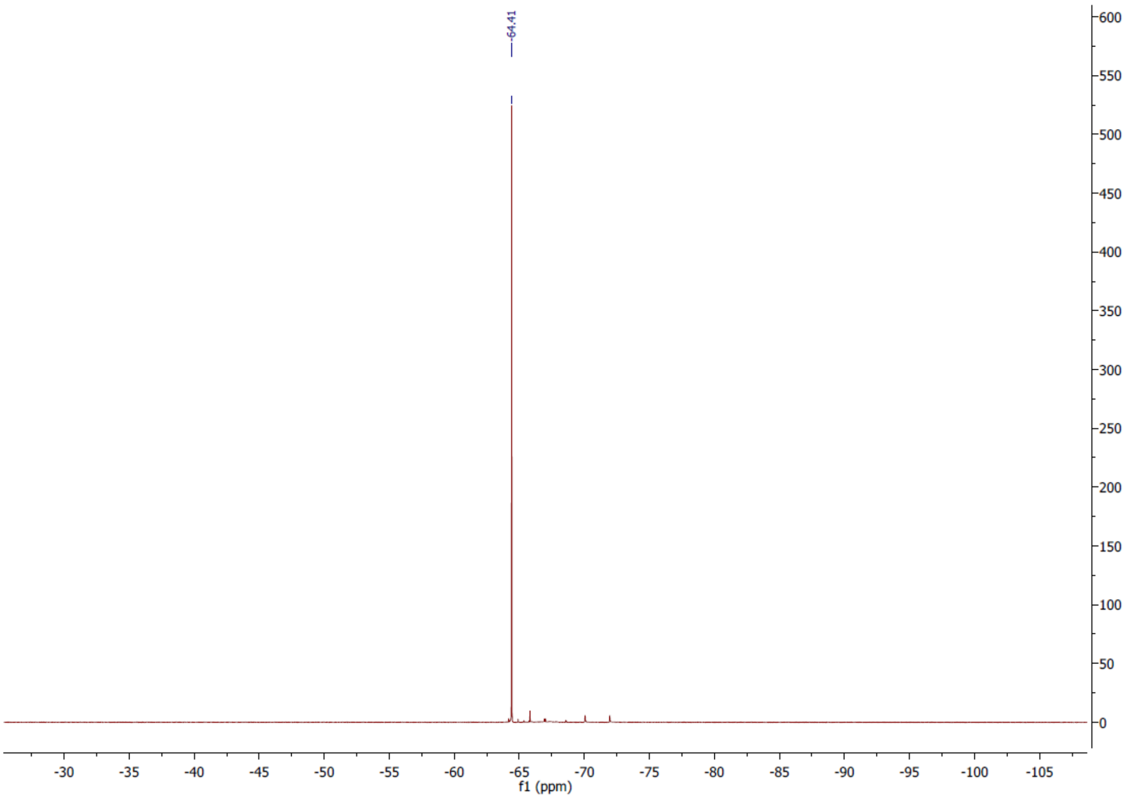
-64.41
f1 (ppm)

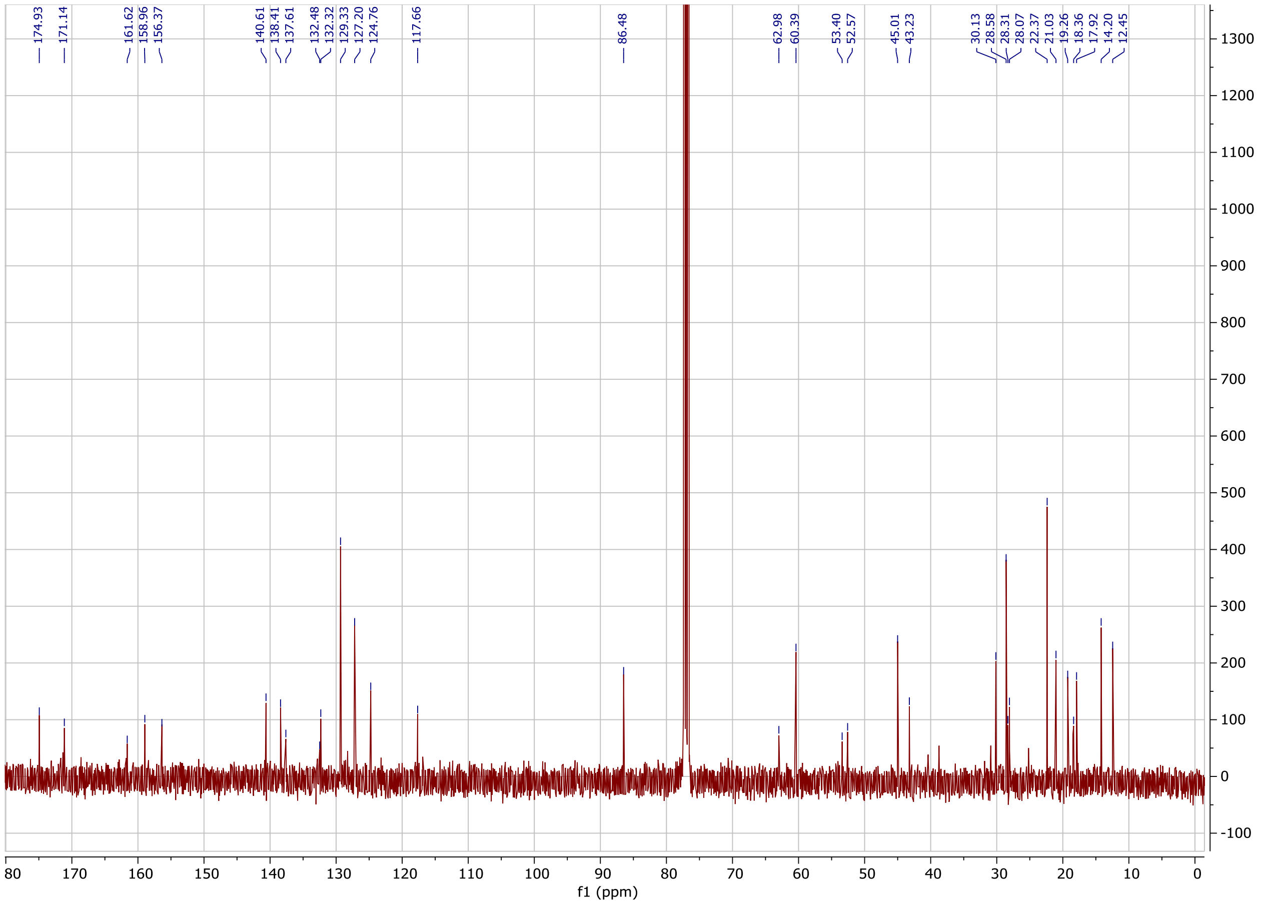

- L.G.S. - Laboratorio Grandi Strumenti - Display Report

Analysis Info

Analysis Name	av_CR_ArgIIBUf.d	Acquisition Date	09/29/23 14:57:07	Operator	Administrator
Sample Name		Method	Copy of $opt1521.MS	Instrument	esquire3000plus
Comment	1mg/ml CH3CNdil 1:100 in CH3CN<br>Richiedente Carola e Paolo				

Acquisition Parameter

Ion Source Type	ESI	Mass Range Mode	Std/Normal	Ion Polarity	Positive	Alternating Ion Polarity	off
Scan Begin	50 m/z	Scan End	1600 m/z	Averages	5 Spectra	Accumulation Time	10 µs
Capillary Exit	149.6 Volt	Skim 1	40.0 Volt	Trap Drive	70.0	Auto MS/MS	off

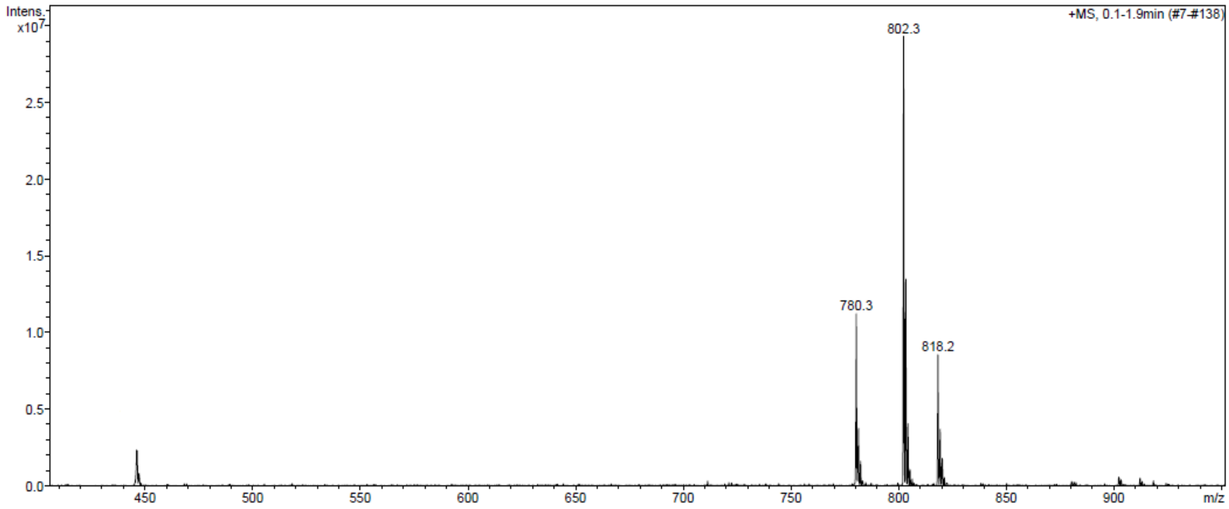

IBU-PAMAM G2-αtfm-βAla-Arg 1

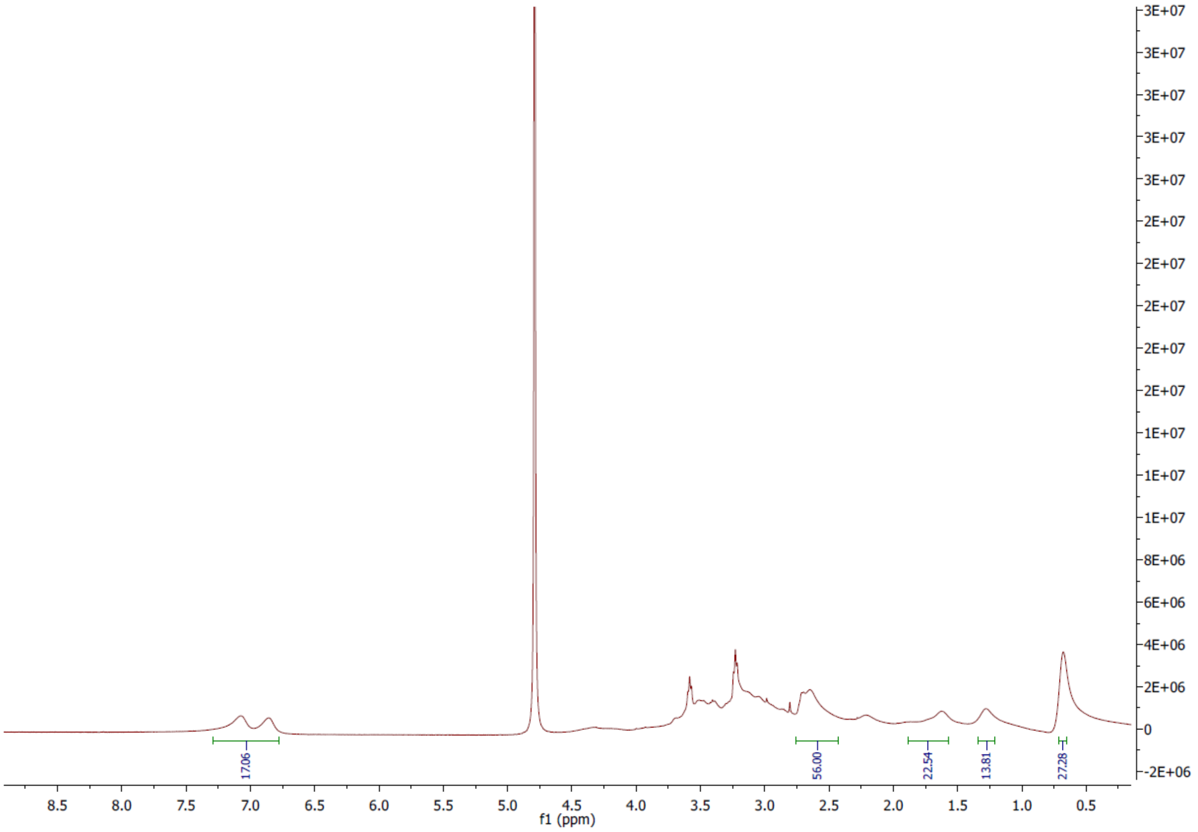

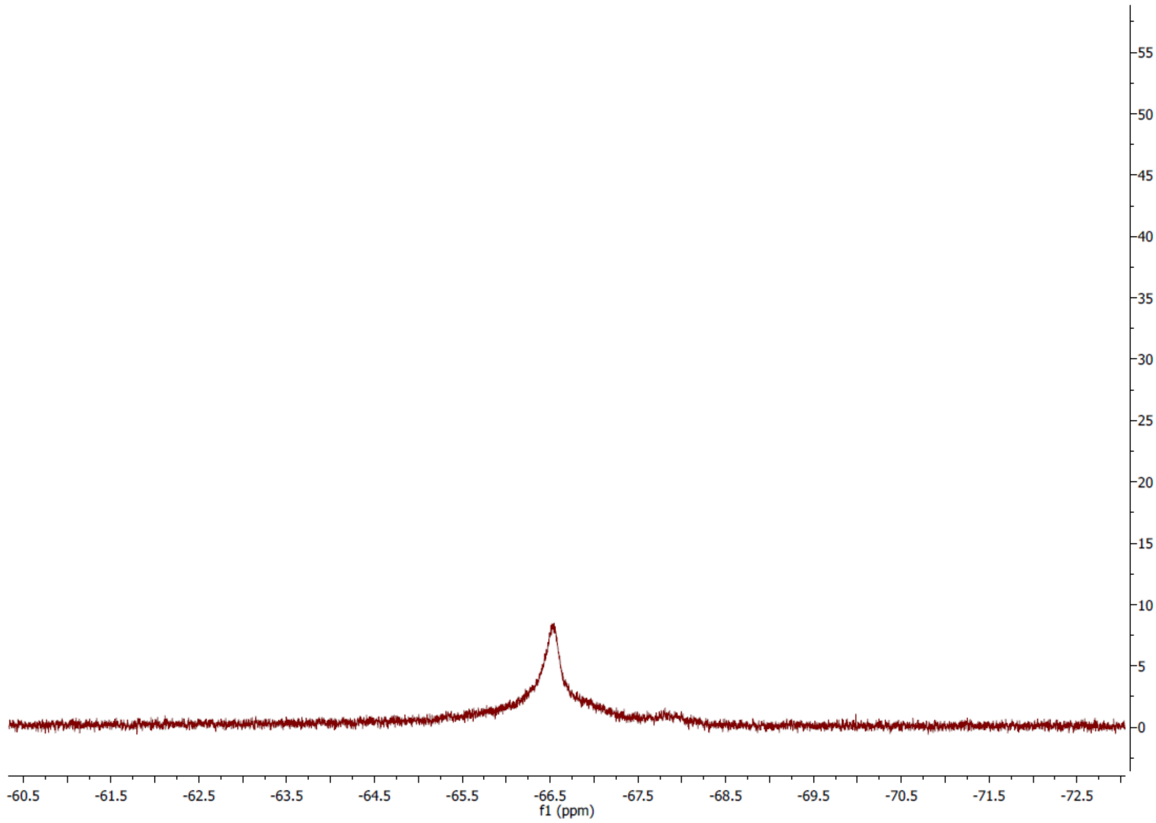

IBU-PAMAM G4-αtfm-βAla-Arg 2

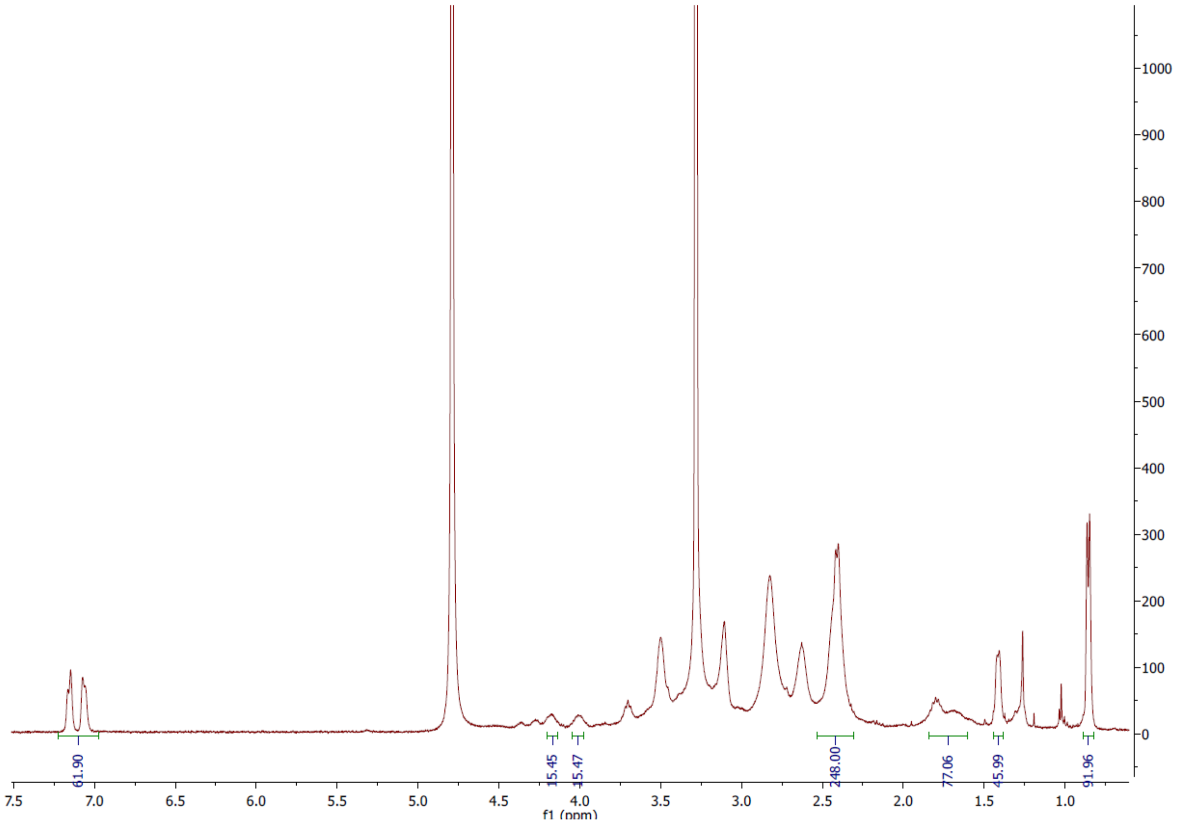

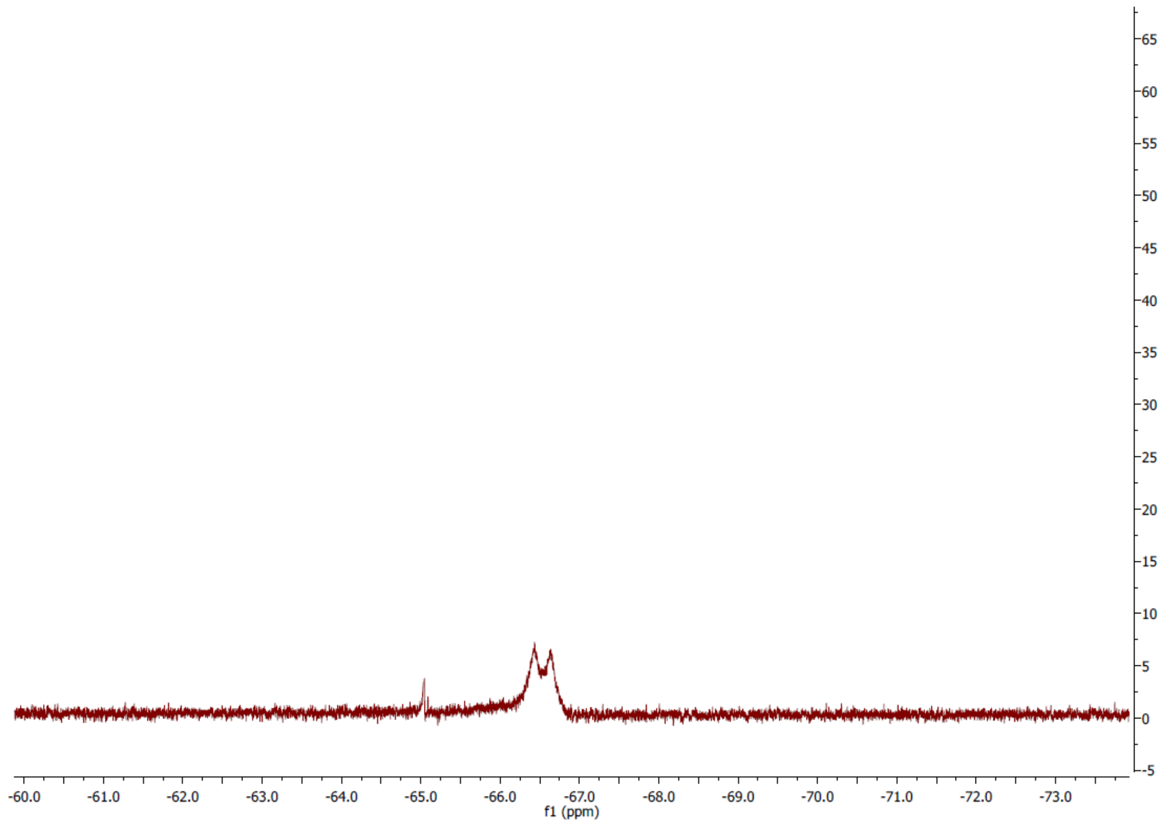

Figure S1. IBU release profile at physiologic pH for the different carriers compared to undecorated PAMAM G4 in 24 hours.

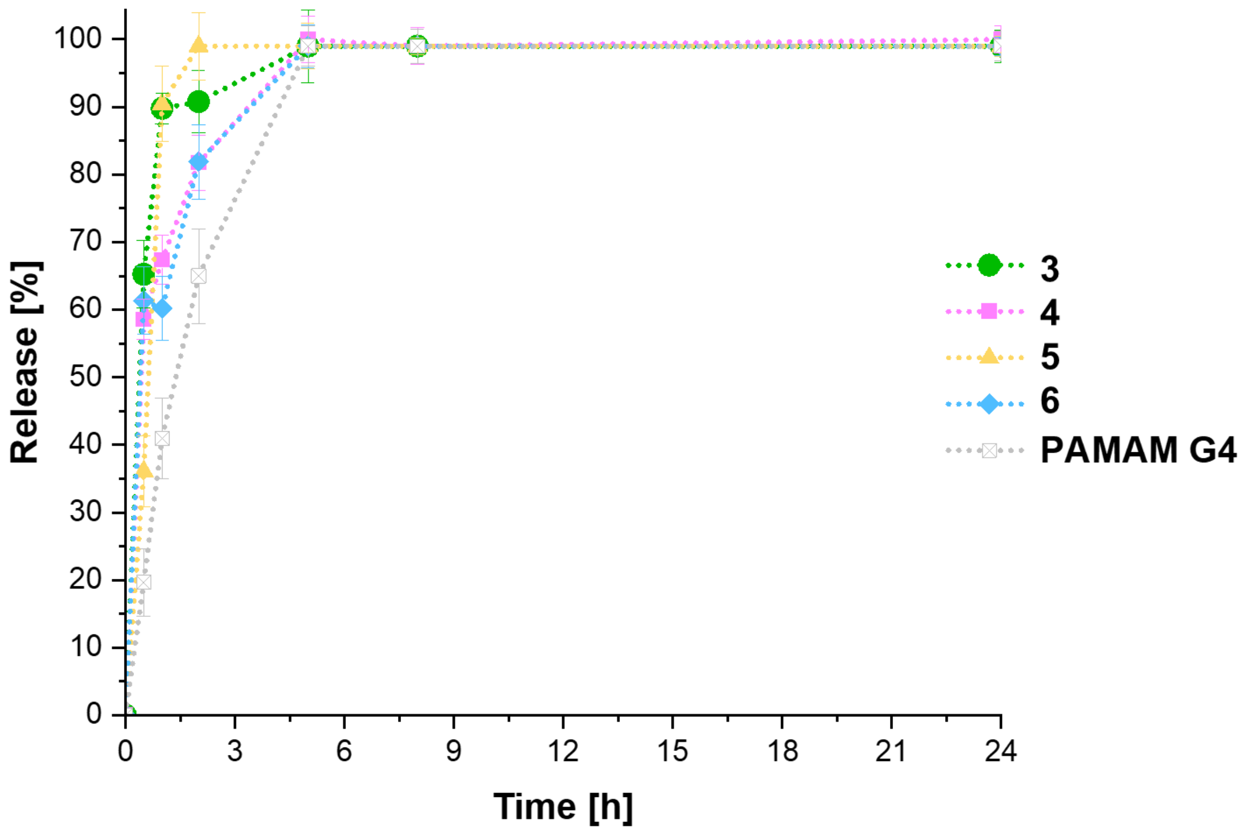

Figure S2. Ibuprofen calibration curve obtained considered 7 IBU dissolved in ACN at known concentrations.

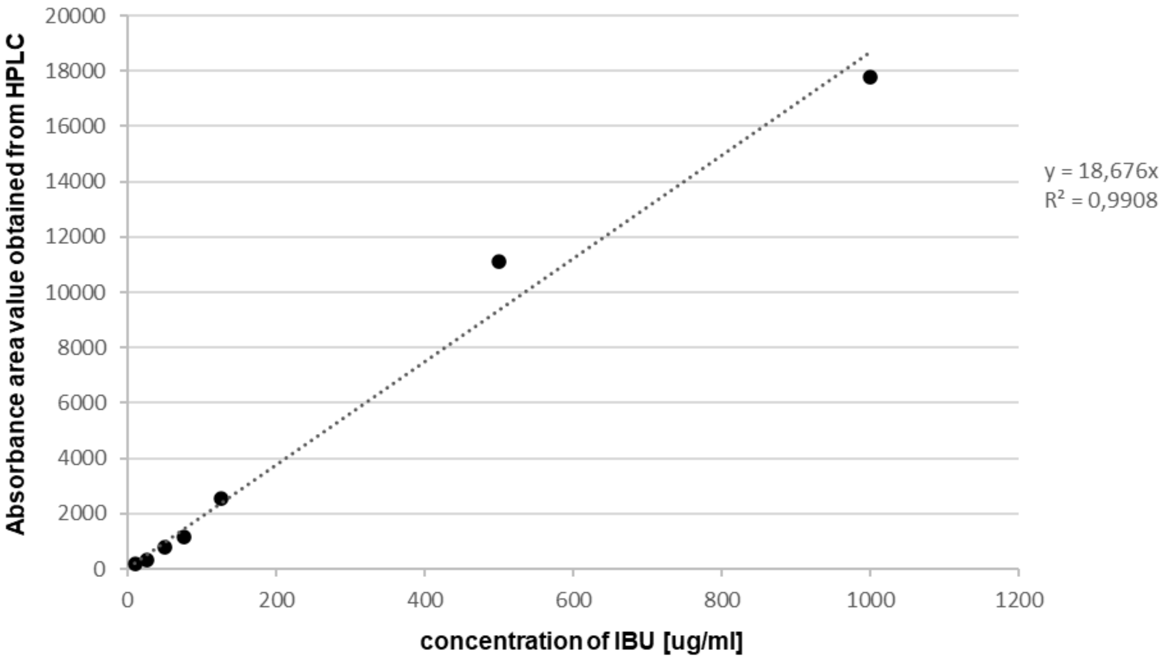

Figure S3. Cytotoxicity of fluorinated PAMAM-Arg conjugates 3-6 after 24h. For negative control (CTR), HUVECs without vehicles were considered. Error bars are standard deviation and statistical significance is **p<0.001 for system 4 and 6 compared to undecorated PAMAM G4 and *p<0.003 for 3 and 5 systems compared to undecorated PAMAM G2.

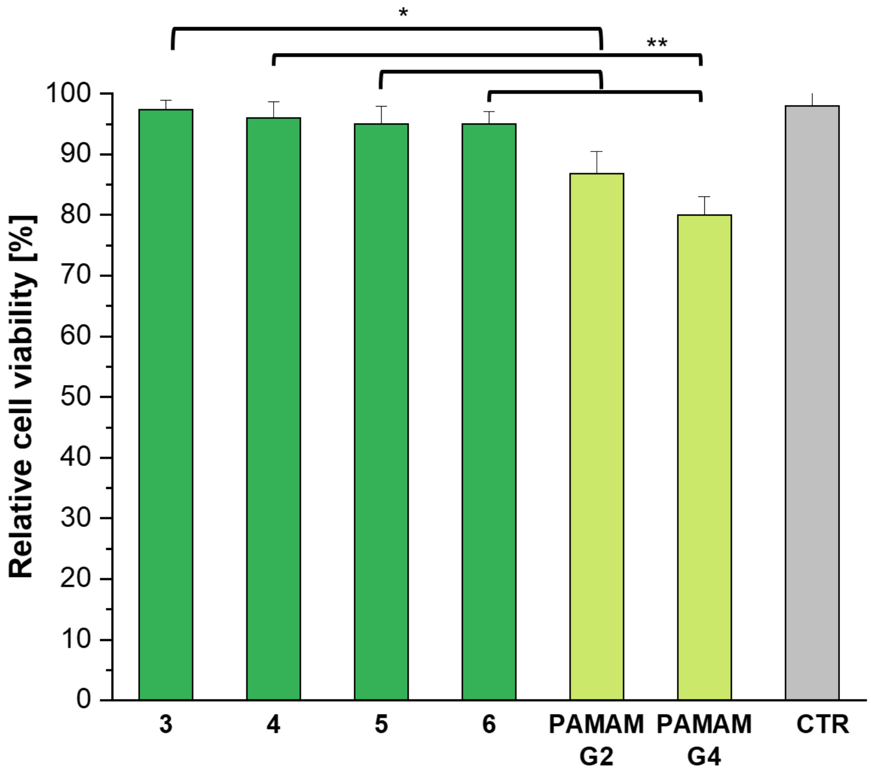

Supplement: Supplementary file 1 — Supplementary file1 (PDF 908 KB) [file 11095_2024_3747_MOESM1_ESM.pdf]
